# Prevalence and burden of HBV co‐infection among people living with HIV: A global systematic review and meta‐analysis

**DOI:** 10.1111/jvh.13217

**Published:** 2019-12-22

**Authors:** Lucy Platt, Clare E. French, Catherine R. McGowan, Keith Sabin, Erin Gower, Adam Trickey, Bethan McDonald, Jason Ong, Jack Stone, Philippa Easterbrook, Peter Vickerman

**Affiliations:** ^1^ Faculty of Public Health & Policy London School of Hygiene & Tropical Medicine London UK; ^2^ NIHR Health Protection Research Unit in Evaluation of Interventions Population Health Sciences Bristol Medical School University of Bristol Bristol UK; ^3^ Humanitarian Public Health Technical Unit Save the Children UK London UK; ^4^ UNAIDS Geneva Switzerland; ^5^ Centre for Disease Control and Prevention Atlanta USA; ^6^ Oxford School of Public Health Nuffield Department of Population Health University of Oxford Oxford UK; ^7^ Oxford University Hospitals NHS Foundation Trust John Radcliffe Hospital Oxford UK; ^8^ Department of Clinical Research London School of Hygiene and Tropical Medicine London UK; ^9^ World Health Organisation Geneva Switzerland

**Keywords:** co‐infection, hepatitis B, HIV, systematic review, viral hepatitis

## Abstract

Globally, in 2017 35 million people were living with HIV (PLHIV) and 257 million had chronic HBV infection (HBsAg positive). The extent of HIV‐HBsAg co‐infection is unknown. We undertook a systematic review to estimate the global burden of HBsAg co‐infection in PLHIV. We searched MEDLINE, Embase and other databases for published studies (2002‐2018) measuring prevalence of HBsAg among PLHIV. The review was registered with PROSPERO (#CRD42019123388). Populations were categorized by HIV‐exposure category. The global burden of co‐infection was estimated by applying regional co‐infection prevalence estimates to UNAIDS estimates of PLHIV. We conducted a meta‐analysis to estimate the odds of HBsAg among PLHIV compared to HIV‐negative individuals. We identified 506 estimates (475 studies) of HIV‐HBsAg co‐infection prevalence from 80/195 (41.0%) countries. Globally, the prevalence of HIV‐HBsAg co‐infection is 7.6% (IQR 5.6%‐12.1%) in PLHIV, or 2.7 million HIV‐HBsAg co‐infections (IQR 2.0‐4.2). The greatest burden (69% of cases; 1.9 million) is in sub‐Saharan Africa. Globally, there was little difference in prevalence of HIV‐HBsAg co‐infection by population group (approximately 6%‐7%), but it was slightly higher among people who inject drugs (11.8% IQR 6.0%‐16.9%). Odds of HBsAg infection were 1.4 times higher among PLHIV compared to HIV‐negative individuals. There is therefore, a high global burden of HIV‐HBsAg co‐infection, especially in sub‐Saharan Africa. Key prevention strategies include infant HBV vaccination, including a timely birth‐dose. Findings also highlight the importance of targeting PLHIV, especially high‐risk groups for testing, catch‐up HBV vaccination and other preventative interventions. The global scale‐up of antiretroviral therapy (ART) for PLHIV using a tenofovir‐based ART regimen provides an opportunity to simultaneously treat those with HBV co‐infection, and in pregnant women to also reduce mother‐to‐child transmission of HBV alongside HIV.

**Abbreviations**
ART: antiretroviral therapy
CHB: chronic hepatitis B
CI: confidence interval
HBsAg: hepatitis B surface antigen
HCC: hepatocellular carcinoma
IQR: interquartile range
MSM: men who have sex with men
PLHIV: people are living with HIV
PWID: people who inject drugs

## 1 INTRODUCTION

Chronic hepatitis B (CHB) infection, defined as persistence of hepatitis B surface antigen (HBsAg), is a major public health problem resulting in an estimated 900 000 deaths in 2015.^1^, ^2^, ^3^, ^4^ Although HBV can be prevented with vaccination, in 2015, there were an estimated 257 million persons chronically infected.^4^ Between 20 and 30% of those with chronic infection develop complications, mainly cirrhosis and hepatocellular carcinoma (HCC).^5^ CHB accounts for 43% of cases of HCC and 40% of cirrhosis, with much higher proportions in lower middle‐income countries,^4^ and 5%‐10% of liver transplants in high‐income countries.^6^ Age is a key determinant of the risk of chronic infection: chronicity is common following acute infection in neonates (around 90%) and young children under the age of 5 years (20–60%), but occurs rarely (<5%) when infection is acquired in adulthood.^7^, ^8^ Worldwide, most persons with CHB were infected at birth or in early childhood.^9^ The highest prevalence of HBsAg (>5%) is in sub‐Saharan Africa, East Asia, parts of Balkans, the Pacific Islands and the Amazon Basin.^10^ Regional variation exists in the epidemiology of HBV: perinatal or horizontal transmission predominates in sub‐Saharan Africa and Asia, whereas in high‐income countries transmission is predominantly via injection drug use and high‐risk sexual behaviours.^9^, ^11^

As PLHIV live longer due to increased access to antiretroviral therapies, liver disease has emerged as a leading cause of death in PLHIV co‐infected with HBV or HCV.^12^, ^13^ Among people with HBsAg, co‐infection with HIV results in higher rates of chronicity and occult HBV (HBV‐DNA positivity in the absence of HBsAg), accelerated liver disease progression, higher liver‐related mortality and decreased treatment response.^14^, ^15^, ^16^, ^17^ Co‐infection with CHB also increases risk of hepatotoxicity from antiretroviral therapy (ART) three‐ to five‐fold,^18^, ^19^ and cross‐resistance between HIV and HBV drugs is common.^20^, ^21^ Fortunately, tenofovir, a drug commonly included in ART regimens, is also the most effective drug for long‐term treatment of HBV, leading to long‐term HBV viral suppression, reversal of cirrhosis and fibrosis, and reduction in HBV‐related mortality.^22^

There is a need to establish the global burden of HBsAg co‐infection among PLHIV, to characterize the most affected populations and geographical regions, and to inform national and regional screening programmes and clinical management. However, to date, no review has estimated the global burden of HBV co‐infection among PLHIV. Existing estimates suggest approximately 10% of PLHIV have chronic hepatitis B or 2‐4 million people, but were based on small numbers of studies with unclear methodology.^17^, ^23^, ^24^ Other reviews have focussed on specific regions^25^ or people who inject drugs (PWID).^11^, ^26^ We therefore undertook a global systematic review of the prevalence and burden of HBsAg in PLHIV.

## 2 METHODS

### 2.1 Search strategy and selection criteria

The systematic review was conducted alongside a companion review examining prevalence and burden of HIV‐HCV antibody co‐infection (consistent with current or past infection) which contains detailed description of the search and synthesis methods.^27^ The review was registered with the PROSPERO prospective register of systematic reviews (CRD42019123388).

In brief, we searched eight databases for material that reported prevalence of HBV and HIV, published between 1 January 2002 and 8 April 2018 following PRISMA guidelines.^28^ The searches were carried out in MEDLINE, EMBASE, CINAHL+, POPLINE, Africa‐wide Information, Global Health, Web of Science, and the Cochrane Library, Index Medicus of the Eastern Mediterranean Region, Index Medicus of the South‐East Asian Region, LILACS and Western Pacific Region Index Medicus. All English and non‐English language sources were included. The search terms used were as follows: ‘HIV OR Human immunodeficiency virus’ and ‘hepatitis‐B OR hepatitis C OR HBV OR HCV’ and ‘prevalen* OR inciden* OR seroprevalen* OR screening OR surveillance OR population* OR survey* OR epidem* OR data collection OR population sample* OR community survey* OR cohort OR cross‐sectional OR longitud* OR follow‐up’. Searches were tailored to the search functionality of each database. The reference lists of articles identified as reviews were screened for additional relevant sources.

We included papers with country‐level estimates of HBsAg co‐infection among an HIV population sample greater than 50, recruited based on their HIV‐positive status or other behavioural characteristics, such as injecting drug use. We excluded editorials or reviews containing no primary data, samples recruited based on their HBsAg status or HIV‐HBsAg‐positive status; studies based on self‐reported HIV or HBsAg status, hospital‐based studies, or in healthcare workers, organ or tissue donors, or from populations with other co‐morbidities such as persons with TB or mental illness, or undergoing interventions that put them at greater risk of co‐infection, including those receiving haemodialysis, those with haemophilia, cancer, cardiovascular disease, other co‐infections, kidney, liver or neurological diseases.

### 2.2 Screening and data extraction

Six reviewers (CF, CM, BM, AT, JS and JO) screened each record with a seventh reviewer (LP) consulted when there was no consensus. Data extracted included the following: study methods; field‐work dates; population; recruitment site; sample size; diagnostic assays used; and prevalence of co‐infection. For approximately 10% of included studies, data were double extracted by a second author (EG) to check the accuracy of data extraction.

### 2.3 Quality assessment

To address concerns of variable quality of studies in previous reviews, we assessed and rated the quality of included studies based on study design and assay quality (Supporting information S1). Studies with larger sample sizes, recruited from multiple sites, recording age and sex or HIV risk factors were scored higher (A); studies with >200 cases from >1 site, with some HIV risk factors recorded but not designed to measure prevalence was scored lower (B); and studies with <200 case from a single site with no risk factors recorded were scored lowest (C). HBsAg assay methods were rated from 0 where no assay type was specified, to up to 3 where a second confirmatory HBsAg assay was done, with or without a neutralisation step. Best estimates were selected for each population group per country based on the highest assay and study design score. Where multiple estimates were available, we applied decision rules to select the best estimate (Supporting information S1).

### 2.4 Classification of countries according to Global Burden of Disease region

Countries were initially grouped according to the 21 Global Burden of Disease regions, and these were then combined into ten regions to be consistent with previous published reviews on HIV, HBsAg and HCV burden.^11^, ^27^, ^29^

### 2.5 Definition of Population groups

We extracted data on risk behaviours associated with HIV and HBV transmission and populations were categorized according to their main HIV‐exposure categories. A general population sample was considered to be low‐risk and included samples of blood donors (unpaid), ante‐natal clinic attendees or general population and household surveys not recruited based on HIV‐positive status. Samples of PLHIV reporting heterosexual transmission as the main risk factor and HIV‐positive pregnant women were grouped together. We categorized study populations as PWID when >75% of the sample had current or past experience of injecting, and as men who have sex with men (MSM) when >50% reported main HIV exposure to be sex with men. The PWID and MSM population categories included studies of both known PLHIV as well as populations recruited based on their risk behaviour but where HIV testing was also done. Two other population groups included the following: high‐risk populations (PLHIV reporting any injecting drug use or sex between men (but ≤75% of the sample for PWID and ≤50% for MSM), sex workers; prison inmates, non‐injecting drug users, STI clinic attendees or a mixed population engaging in sexual and/or injecting risk behaviours but with ≤75% of the sample injecting); and children and young people (aged between two months and 17 years).

### 2.6 Data synthesis

We report HIV‐HBsAg co‐infection prevalence among six population groups (general population, heterosexual and pregnant women, PWID, MSM, children and young people, and other high‐risk populations) by country and region, reporting the best estimate and range for each country from all studies. Global and regional estimates of prevalence were derived from the median of the ‘best’ estimates for that region and presented alongside the interquartile range (IQR) of the best estimates. Data were entered into R (R Foundation for Statistical Computing, Vienna, Austria) to generate maps presenting country‐level HIV‐HBV co‐infection prevalence estimates.

Across these six populations, we also synthesized estimates of HBsAg prevalence in PLHIV and HIV‐negative populations where samples were recruited based on population characteristic rather than known HIV status and undertook a meta‐analysis of the odds of being HBsAg positive among HIV‐positive populations compared to HIV‐negative populations stratified by population group. A standard correction of 0.5 was added to all zero prevalence estimates using STATA 14.1 (Stata Corp). Odds ratios were calculated through a Mantel‐Haenszel method with a random‐effect model. Meta‐analyses of sub‐groups are presented as forest plots including the odds ratio and 95% confidence interval (CI).

Finally, we report global and regional estimates of burden of HBsAg co‐infection among PLHIV in 2017. Using number of persons with HIV infection by country and region estimated through the Spectrum model and reported by the Joint United Nations Programmes on HIV/AIDS (UNAIDS),^30^, ^31^ we applied median best estimates of HBsAg co‐infection prevalence among PLHIV not exposed via injecting drug use from all surveys included from the literature search for MSM, general population and HIV‐positive samples of pregnant women or those heterosexually exposed by regions, and overall. Median best estimates of HBsAg co‐infection prevalence among HIV‐positive PWID were applied to the distribution of PLHIV exposed via injecting drug use, as estimated by UNODC, across regions and overall.

### 2.7 Role of the funding source

The WHO commissioned this review for the purpose of informing the update of the WHO guidelines on testing for viral hepatitis.^22^, ^23^ The funder contributed to the data collection, analysis, interpretation and writing of the review. All authors had full access to the study data and share final responsibility for the findings submitted for publication. The full dataset and statistical source code used to generate estimates and select best estimates is available from the corresponding author on request.

## 3 RESULTS

**Figure 1:**
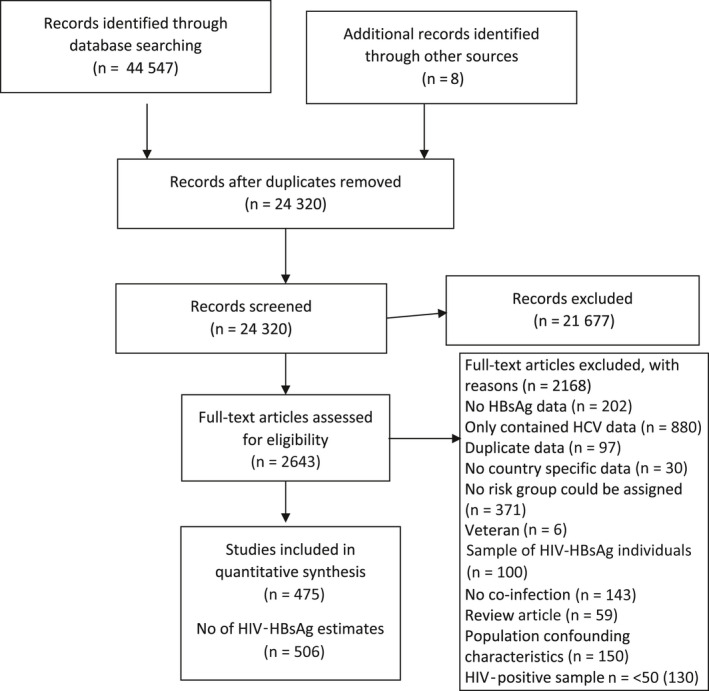
Flow chart of included studies

Figure 1 summarizes the flow chart for the identification and selection of studies. From an overall 44 547 publication references, 475 papers/studies met the inclusion criteria resulting in 506 estimates of the prevalence of HIV‐HBsAg co‐infection across six population groups (general populations, PLHIV heterosexual and pregnant women, PWID, MSM, other high‐risk populations and children).

### 3.1 Availability of studies and estimates by region and population group

Overall, 80 (41.0%) of 195 countries had estimates. One‐third (n = 28) of these countries were from sub‐Saharan Africa and 26.3% (n = 21) from Western/Central/Eastern Europe, with these regions having the highest proportion of countries with data (28/45 62.2% and 21/54 38.9%, respectively). The number and proportion of countries with estimates from other regions were as follows: North Africa and Middle East (7/21 33.3%); Latin America (9/36 25.0%); East Asia (2/2 100%); South and South‐East Asia (8/18 44.4%); Asia Pacific and Australasia (3/17 17.6%); and North America (2/2 100%).

Of the 506 co‐infection prevalence estimates, 45 were from general population samples (8.9% of estimates), 90 from PLHIV populations who were either pregnant women or where heterosexual transmission was the primary exposure (17.8% of estimates), 36 among PWID (7.1%), 70 among MSM (13.8%), 22 among children and young people, and 243 (48.0%) from high‐risk populations (202 from mixed PWID and MSM populations, five among sex workers, seven among prisoners and 29 from other high‐risk populations).

### 3.2 Rating of study quality and assay method

Very few estimates were rated ‘A’ in study design quality (15/506), based on large multisite surveys. 63.8% (323/506) of studies were rated ‘B’ in study design quality, based on data from more than one site with a sample size >200, and 33.2% as ‘C’ (168/506). Two‐fifths (n = 207) of estimates provided no information on type of HBsAg assay (rated 0), 23 estimates were based on a rapid test (rated 1), 218 used an HBsAg assay (any generation) with no confirmatory HBsAg assay (rated 2), and the remaining 58 used an HBsAg assay (any generation) with a confirmatory HBsAg assay (rated 3). Taking both assay and study design together, the highest rated study was among PWID in Vietnam^33^ (rated A3), with five studies (1.0%) having the next highest rating (A2). Most estimates were categorized as B0 (28.1%), B2 (27.3%), followed by C2 (14.8%) and C0 (11.1%). This information is summarized in Table S2.

### 3.3 Prevalence of HIV‐HBsAg co‐infection by population group

#### 3.3.1 General population samples

**Figure 2:**
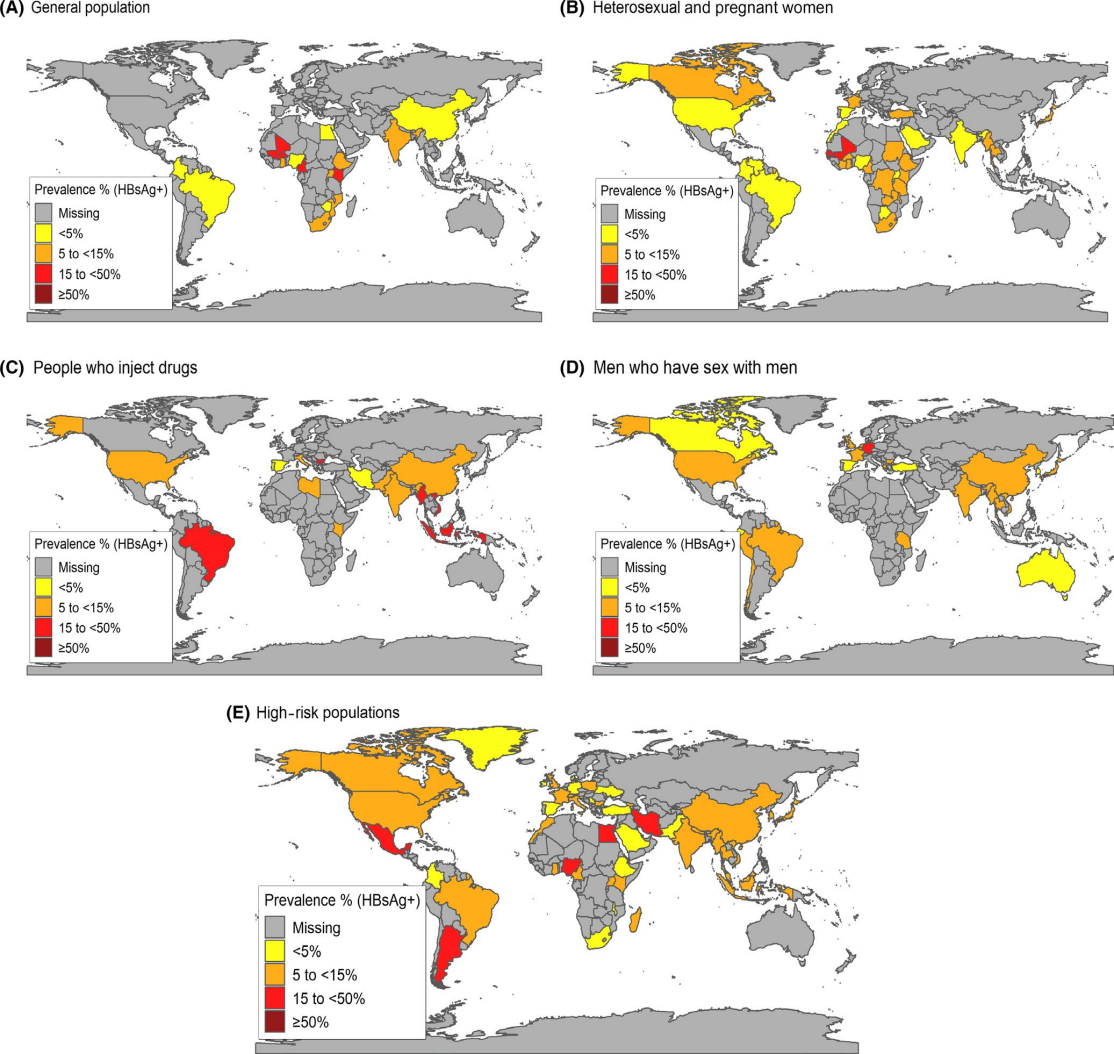
Best estimates of hepatitis B Surface Antigen co‐infection prevalence among samples of A, General population B, Heterosexual and pregnant women. C, People who inject drugs; D, Men who have sex with men; and E, Other high‐risk individuals.

The mid‐point prevalence of HBsAg co‐infection among 45 general population samples testing positive for HIV was 7.4% (IQR 1.4%‐15.7%) with country‐level prevalence estimates shown in Figure 2A. The highest prevalence was from West and Central Africa at 16.4% compared to 4.4% and 8.8% from South and East Africa, respectively. Very low prevalence was reported from East Asia (0.4%, one study) and Latin America (0.9%). There were no general population studies from Europe, North America or Asia Pacific/Australasia. All estimates are summarized in Table 1 with global prevalence maps shown in Figure 2.

**Table 1:** Summary of global HIV‐HBsAg co‐infection prevalence estimate in general population sample, heterosexual and pregnant PLHIV, PWID and MSM

	General Population						Heterosexual and pregnant women						PWID						MSM						
	Total studies		Best estimate				Total studies		Best estimate				Total studies		Best estimate				Total studies		Best estimate				
Country	n	Range^b^	%	S	n	Year	n	Range^b^	%	S	n	Year	n	Range^b^	%	S	n	Year	N	Range^b^	%	S	n	Year	
**West and Central Africa**																									
Burkina Faso^68^, ^69^, ^70^	2	0.5‐17.0	17.0	B3	761	2010	1	6.1	6.1	C3	115	2009													
Cameroon^71^, ^72^, ^73^, ^74^, ^75^, ^76^, ^77^, ^78^	2	4.2‐17.3	17.3	B3	301	2013	6^c^	8.3‐14.6	11.8	B3	212	2010													
Cote D'Ivoire^79^, ^80^							2^c^	9.0‐12.7	12.7	C2	495	2006													
Congo^81^							1	7.7	7.7	B2	209	2008													
Equatorial Guinea^82^	1	15.7	15.7	B3	230	2013																			
Gambia^83^							1	12.2	12.2	C0	572	2009													
Ghana^84^, ^85^, ^86^, ^87^, ^88^, ^89^	4	2.4‐18.7	6.0	C2	168	2007	2	10.8‐11.0	11.0	C2	155	2010													
Guinea‐Bissau^90^							1	16	16.0	B1	576	2011													
Mali^91^, ^92^	1	25.3	25.3	C2	518	2002	1	22	22.0	B2	242	2004													
Nigeria^93^, ^94^, ^95^, ^96^, ^97^, ^98^, ^99^, ^100^, ^101^, ^102^, ^103^, ^104^, ^105^, ^106^, ^107^, ^108^, ^109^, ^110^, ^111^, ^112^	8	0.0‐19.8	1.3	B2	174	2016	12^c^	2.0‐11.9	4.2	B2	2391	2011													
Senegal^113^							1	16.8	16.8	C2	363	2002													
**Total** a	18	6.0‐17.3	16.4				28	7.7‐16.0	12.0																
**South Africa**																									
Botswana^114^, ^115^							2	0.0‐5.3	0.0	B2	1995	2006													
Lesotho^116^							1	5.5	5.5	B2	205	2007													
South Africa^117^, ^118^, ^119^, ^120^, ^121^, ^122^	1	7.4	7.4	B3	215	2009	5^c^	2.1‐7.4	7.4	B3	189	2013													
Zimbabwe^123^	1	1.4	1.4	C2	74	2005																			
**Total** a	2	1.4‐7.4	4.4				8	0.0‐7.4	5.5																
**East Africa**																									
Comoros^124^							1	8	8.0	C0	50	2002													
Djibouti^125^	1	9.7	9.7	C3	175	2000																			
Ethiopia^126^, ^127^, ^128^, ^129^, ^130^, ^131^, ^132^, ^133^	4	0.7‐61.4	7.9	B2	101	2013	4	3.0‐6.1	5.0	C3	400	2011													
Kenya^36^, ^134^, ^135^, ^136^, ^137^, ^138^	1	20.9	6.8	B1	267	2007	4	5.2‐6.9	6.1	B2	378	2007	1	13.9	13.9	C2	72	2010							
Malawi^139^, ^140^, ^141^							3^c^	5.0‐16.9	8.7	C3	309	2009													
Mozambique^142^, ^143^	2	10.1‐13.8	13.8	C0	58	2009																			
Rwanda^144^, ^145^	1	5.7	5.7	C2	384	2001	1^c^	2.4	2.4	C3	85	2004													
Tanzania^146^, ^147^							1^c^	6.2	6.2	C0	17 539	2011							1	9.2	9.2	B0	65	2007	
Uganda^145^, ^148^, ^149^, ^150^, ^151^, ^152^	4	4.6‐8.3	6.0	B2	72	1999	2^c^	4.6‐4.9	4.9	C3	164	2004													
Zambia^153^							1	9.9	9.9	C2	323	2008													
**Total** a	13	6.0‐13.8	8.8				17	4.9‐8.4	6.1				1	13.9	13.9				1	9.2	9.2				
**N Africa & Middle East**																									
Egypt^154^	1	3.4	3.4	C2	59	2015																			
Iran (Islamic Republic of)^35^, ^155^, ^156^, ^157^, ^158^													5	2.1‐44.2	2.1	B3	888	2007							
Lebanon																									
Libya^159^													1	5.1	5.1	B0	294	2010							
Morocco^160^							1^c^	2.4	2.4	C2	1120	2015													
Saudi Arabia^161^							1	3.4	3.4	C1	234	2010													
Sudan^162^, ^163^							2	11.7‐14.5	11.7	B3	358	2012													
Total^a^	1	3.4	3.4				4	2.4‐11.7	3.4				6	2.1‐5.1	3.6										
West, Central and East Europe																									
Belgium^164^, ^165^																			3	0.0‐6.1	6.1	B0	3081	2009	
Bulgaria^166^													1	16.9	16.9	B2	359	2011	1	9.7	9.7	B2	1087	2011	
Denmark																									
France^167^, ^168^, ^169^, ^170^, ^171^							2^c^	6.0‐6.9	6.9	B0	6548	2013							2	5.0‐8.8	8.8	B2	2351	2002	
Georgia																									
Germany^169^, ^172^, ^173^, ^174^																			4	1.7‐25.4	25.4	B3	1843	2012	
Greece^175^, ^176^																			2	6.0‐6.5	6.0	A2	1729	2003	
Ireland																									
Italy^177^, ^178^													2	3.5‐7.0	7.0	C2	173	2006							
Moldova													1	11.0	11.0	C3	113	2009							
Netherlands^179^, ^180^, ^181^, ^182^							1^c^	4.9	4.9	B0	1546	2008							2	5.2‐7.7	5.2	A2	12 800	2012	
Poland																									
Portugal^183^													1	4.1	4.1	C0	343	2005							
Romania																									
Spain^184^, ^185^, ^186^, ^187^, ^188^							1	2.3	2.3	B0	741	2006	1	3.9	3.9	B0	821	2015	4	4.3‐10.9	4.3	B2	392	2011	
Serbia																									
Slovenia																									
Switzerland																									
Turkey^189^, ^190^, ^191^							2	7.1‐11.4	11.4	C3	70	2007							1	4.0‐4.0	4.0	B0	55	2009	
United Kingdom^192^, ^193^																			2	2.1‐5.3	5.3	A0	25 486	2012	
Ukraine																									
**Total** a							6	4.9‐7.1	5.9				6	4.1‐11.0	7.0				21	5.2‐8.8	6.0				
**East Asia**																									
China^34^, ^194^, ^195^, ^196^, ^197^, ^198^, ^199^	1	0.4	0.4	C0	275	2017							5	1.1‐59.6	11.8	B3	498	2014	1	14.2‐14.2	14.2	B0	532	2010	
China, province of Taiwan^200^, ^201^, ^202^, ^203^, ^204^, ^205^, ^206^, ^207^, ^208^, ^209^, ^210^, ^211^, ^212^, ^213^, ^214^, ^215^, ^216^, ^217^							1	12.8	12.8	B2	105	2005	6	17.4‐20.0	18.9	A2	301	2010	13	3.5‐22.0	3.5	C3	523	2012	
**Total** a	1	0.4	0.4				1	12.8	12.8				11	11.8‐18.9	15.4				14	3.5‐14.2	8.8				
**South and South‐East Asia**																									
Cambodia																									
India^218^, ^219^, ^220^, ^221^, ^222^, ^223^, ^224^, ^225^, ^226^, ^227^, ^228^, ^229^, ^230^, ^231^, ^232^, ^233^, ^234^, ^235^	6	0.0‐8.3	8.3	B2	121	2017	8	0.0‐7.1	3.4	B2	3142	2012	2	11.9‐12.9	12.9	B2	595	2014	2	3.2‐7.9	7.9	B2	1178	2002	
Indonesia^236^, ^237^													2	7.0‐17.6	17.6	B3	74	2009							
Malaysia																									
Myanmar^34^, ^238^, ^239^							2	8.0‐9.0	8.0	C1	122	2009	2	10.2‐41.9	41.9	B2	86	2009	1	13.4	13.4	C0	176	2012	
Pakistan^240^, ^241^													2	6.0‐37.0	6.0	C3	100	2013							
Thailand^242^, ^243^, ^244^							2	9.0‐11.9	9.0	B2	416	2008							1	5.6	5.6	B2	215	2014	
Vietnam^33^, ^245^, ^246^														2	9.4‐16.3	16.3	A3	849	2009	1	13.2	13.2	C0	153	2014
**Total** a	6	8.3	8.3				12	3.6‐9.2	5.9				10	12.9‐17.6	16.3				5	6.7‐13.3	10.6				
**Asia Pacific & Australasia**																									
Australia^247^, ^248^, ^249^, ^250^																			4	3.4‐6.3	4.9	B2	1719	2001	
Japan^251^, ^252^, ^253^, ^254^, ^255^, ^256^							1	5.4	5.4	B2	166	2002							6	6.3‐17.9	7.2	B3	817	2013	
South Korea^257^																			1	5	5.0	B0	541	2013	
**Total** a							1	5.4	5.4										11	4.9‐7.2	5.0				
**Latin America (Central, South America & Caribbean)**																									
Argentina																									
Brazil^258^, ^259^, ^260^, ^261^, ^262^, ^263^, ^264^, ^265^, ^266^, ^267^, ^268^, ^269^, ^270^, ^271^	3	0.5‐1.0	0.5	B2	186	2012	6^c^	0.5‐3.3	2.3	C3	130	2008	1	27.3	27.3	B2	205	2003	5	2.9‐31.0	8.2	B2	170	2000	
Chile^272^																			1	6.1	6.1	B0	395	2007	
Colombia^273^, ^274^, ^275^	1	1.2	1.2	C3	247	2009	2	2.9‐3.3	3.3	B2	275	2010													
Ecuador^276^																			1	4.0	4.0	C0	50	2012	
Haiti^277^							1^c^	2.4	2.4	B3	123	2012													
Peru^278^																			1	9.5	9.5	B0	338	2003	
Venezuela^279^, ^280^							2	3.1‐11.8	3.1	C3	418	2008													
**Total** a	4	0.5‐1.1	0.9				11	2.4‐3.2	2.8				1	27.3	27.3				8	5.1‐8.9	7.2				
**North America**																									
Canada^154^, ^281^							1^c^	5.6	5.6	C0	142	2010							1	2.8	2.8	B2	294	2012	
USA^282^, ^283^, ^284^, ^285^, ^286^, ^287^, ^288^, ^289^, ^290^, ^291^							1^c^	2.9	2.9	B2	1500	1995	1	7.0	7.0	C0	3987	2001	9	1.8‐9.3	5.5	B2	816	2006	
**Total** a							2	2.9‐5.6	4.2				1	7.0	7.0				10	2.8‐5.5	4.2				
**Global total** a	45	1.4‐15.7	7.4				90	3.4‐11.0	6.1				36	6.0‐16.9	11.8				70	5.0‐9.2	6.1				

Abbreviation: S, Study quality score.

^a^ Totals are derived from median of best estimates scored with interquartile range of best estimates.

^b^ Range is presented for country‐level estimates and interquartile range for regional and global totals. All best estimates are selected according to the decision rules in Text Box S2.

^c^ Denotes prevalence (total) derived from samples of PLHIV pregnant women among the population group PLHIV (heterosexual and pregnant women) including: Cameroon (2) 9.3%,^74^ 14.6%^73^; Cote D’Ivoire (1) 9.0%^80^; Nigeria (1) 4.2%^105^; South Africa (3) 6.2%,^120^ 3.4%,^118^ 2.1%^117^; Malawi (1) 8.7%^141^; Rwanda (1) 2.4%^145^; Tanzania (1) 6.2%^147^; Uganda (1) 4.9%^145^; Brazil (5) 1.9%,^261^ 0.5%,^266^ 2.3%,^260^ 0.9%,^262^ 1.2%^268^; USA (1) 2.9%^290^; Canada (1) 5.6%^154^; Haiti (1) 2.4%^277^; France (2) 6.0%,^170^ 6.9%^168^; the Netherlands (1) 4.9%,^180^ and Morocco (1).

#### 3.3.2 Heterosexual and pregnant women

The mid‐point prevalence of HBsAg co‐infection among 90 studies in PLHIV heterosexual or pregnant women was 6.1% (IQR 3.4%‐11.0%) with country‐level prevalence estimates shown in Figure 2B. Prevalence was higher in East Asia (12.8%, one study), West and Central Africa (12.0%), and lowest in Latin America (2.8%), North Africa and the Middle East (3.4%) and North America (4.2%). Among this population, there were 23 estimates for pregnant women from 15 countries (see footnote to Table 1 for country‐specific estimates). The mid‐point prevalence of HBsAg co‐infection among 67 studies in heterosexual PLHIV was 8.0% (IQR 5.0%‐11.8%) compared to 4.6% (2.4%‐5.9%) among PLHIV pregnant women (data not shown).

#### 3.3.3 People who inject drugs

The mid‐point prevalence among PWID based on 36 studies was 11.8% (IQR 6.0%‐16.9%) with country‐level prevalence estimates shown in Figure 2C. The highest prevalence was observed in Latin America (27.3%, one study), South and South‐East Asia (16.3%), East Asia (15.4%), and the lowest in North Africa and the Middle East (3.6%). Some single studies reported very high prevalence, including China (59.6%),^34^ Iran (44.2%)^35^ and Myanmar (41.9%).^34^ There was only one study from sub‐Saharan Africa (Kenya) which reported a prevalence of 13.9%.^36^

#### 3.3.4 Men who have sex with men

The mid‐point prevalence among MSM was 6.1% (IQR 5.0%‐9.2%) based on 70 studies with country‐level prevalence estimates shown in Figure 2D. Prevalence was highest in South and South‐East Asia (10.6%), West and Central Africa (9.2%, one study), followed by East Asia (8.8%) and Latin America (7.2%). Lower prevalences of around 4%‐6% were reported from Asia Pacific/Australasia, Europe and North America. There were no studies from East Africa, South Africa or North Africa and the Middle East.

#### 3.3.5 Other high‐risk groups

In addition to the main HIV‐exposure categories, there were 243 estimates from samples of PLHIV engaging in mixed sexual and/or injecting risk behaviours (Table S3) with country‐level prevalence estimates shown in Figure 2E. The point prevalence was 6.4% (IQR 4.3%‐9.6%) and was similar across the regions, except for East Asia (15.6%), and West and Central Africa (10.6%).

#### 3.3.6 Children and young people

There were 22 estimates of HIV‐HBsAg co‐infection among children and young people from 13 countries (Table S3). The mid‐point prevalence was 6.8% (IQR 2.5‐10.0). Prevalence ranged from 2% to 20% across six estimates from Nigeria^37^, ^38^, ^39^, ^40^, ^41^, ^42^ and between 0% and 20.5% in South Africa.^43^, ^44^, ^45^, ^46^ Extremely high prevalence (43%‐46%) was observed in Romania in two studies^47^, ^48^ where HIV infection was acquired nosocomially prior to 1995. A high prevalence (32.6%) was also found in Thailand among young people perinatally infected with HIV (mean age 14 years).^49^ Prevalence was lower (~2%) among samples in India, Malawi, Poland, Spain and the United States,^50^, ^51^, ^52^, ^53^, ^54^ among HIV‐positive paediatric patients. Prevalence was higher in Benin (9.6%), Rwanda (6.8%), Tanzania (7%) and Zambia (10.4%) among children <16 years recruited through HIV clinics.^48^, ^55^, ^56^, ^57^ HIV‐exposure categories were not consistently reported.

#### 3.3.7 Odds of HBsAg positivity among PLHIV compared to HIV‐negative persons

**Figure 3:**
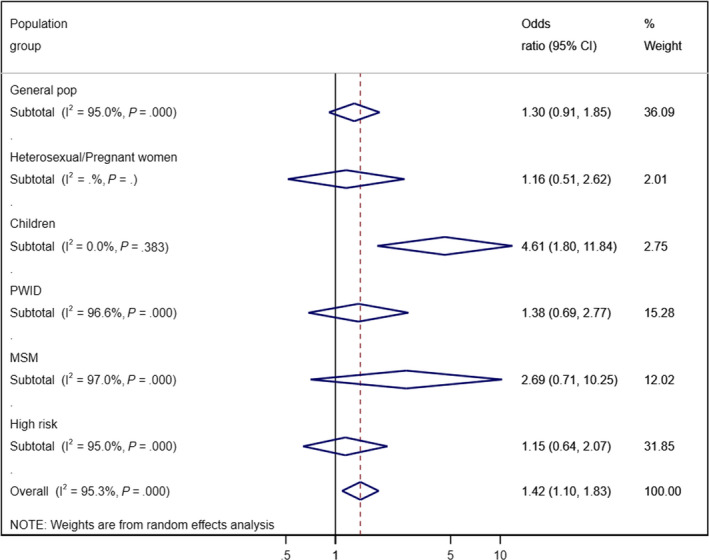
Forest plot showing meta‐analysis of odds of HBsAg infection in HIV‐positive populations versus HIV‐negative populations

Overall, when we compared HBsAg estimates from 25 598 HIV‐positive with 286 121 HIV‐negative individuals, we found increased odds for HBsAg positivity among all HIV‐positive population groups compared to HIV‐negative populations (OR = 1.42; 95% CI = 1.10‐1.83) although there was a high degree of heterogeneity (I squared = 95.3%, *P* < .001). Odds of HBsAg were highest among HIV‐positive children (OR = 4.61; 95% CI = 1.80‐11.84), and there was a borderline statistically significant increase among MSM (2.69; 95% CI 0.71‐10.25), but not with other population groups. This is summarized in Figure 3 and Table S4.

#### 3.3.8 Global burden of HBV co‐infection among PLHIV

Based on 2017 UNAIDS estimates of the number of PLHIV and PWID infected with HIV by region, we estimate that there are 2 653 300 (IQR = 1 970 300‐4 238 300) cases of HBsAg co‐infection among PLHIV globally. Only 3% of cases (n = 142 000; IQR = 124 400‐154 700) are among HIV‐positive PWID. This equates to a global prevalence of HBsAg co‐infection among PLHIV of 7.6% (IQR 5.6%‐12.1%). Sub‐Saharan Africa has the largest burden of HIV‐HBsAg co‐infection representing 69% of the total number of cases, followed by South‐East Asia (12%) and Latin America (6%). All other regions account for <5% each (Table 2).

**Table 2:** Global estimates of HBsAg infection among People living with in 2017 HIV by global burden of disease region

Region	PLHIV (excluding PWID)			PLHIV PWID				Total PLHIV^a^		
	PLHIV	HBsAg Co‐infection		PLHIV		HBsAg Co‐infection		PLHIV	HBsAg Co‐infection	
	n	Median Prevalence (IQR)^b^	Estimates (IQR)	n	% PWID^c^	Median Prevalence (IQR)^f^	Estimates (IQR)	n	Estimates (range)	Region
Africa (South, West, East, Central)	23 965 000	8.0 (5.9‐13.3)	1 905 200 (1 405 400‐3 177 800)	41 700	0.2%	13.9^e^	5800^e^	24 006 700	1 911 000 (1 411 200‐3 183 600)	69%
North Africa and Middle East	182 040	3.4 (2.9‐7.6)	6200 (5200‐13 800)	37 100	17%	3.6 (2.1‐5.1)	1300 (800‐1900)	219 140	7500 (6000‐15 700)	1%
Europe (West, Central)	839 730	6.0 (4.9‐8.8)	50 400 (41 300‐73 900)	32 000	4%	5.5 (4.0‐12.0)	1800 (1300‐3800)	871 730	52 200 (42 600‐77 700)	2%
Eastern Europe/ CAR	979 140	6.1 (4.0‐9.9)^d^	59 700 (39 200‐96 900)	451 000	32%	11.0^e^	49 600^e^	1 430 140	109 300 (88 800‐146 500)	4%
East Asia	769 500	8.1 (1.9‐13.5)	62 400 (14 700‐103 700)	162 300	17%	15.4 (11.8‐18.9)	25 000 (19 200‐30 700)	931 800	87 400 (33 900‐134 400)	3%
South and South‐East Asia	3 729 800	8.1 (6.7‐11.1)	303 300 (251 400‐414 000)	322 000	8%	16.3 (12.9‐17.6)	52 300 (41 500‐56 600)	4 051 800	355 600 (292 900‐470 600)	12%
Western Pacific (Asia Pacific, Australasia)	118 500	5.2 (4.9‐6.3)	6200 (5900‐7500)	1200	1%	11.8 (6.0‐16.9)^d^	100 (100‐200)	119 700	6300 (6000‐7700)	0%
Latin America (South, Central America, Caribbean)	2 108 900	3.2 (2.3‐6.1)	67 200 (48 500‐128 600)	5000	0.2%	27.3^e^	1400^e^	2 113 900	68 600 (49 900‐130 000)	6%
North America	1 209 700	4.2 (2.8‐5.6)	50 700 (34 300‐67 400)	66 300	5%	7.0^e^	4700^e^	1 276 000	55 400 (39 000‐72 100)	4%
Total	33 902 310	6.1 (4.0‐9.9)	2 511 300 (1 845 900‐4 083 600)	1 118 600	3%	11.8 (6.0‐16.9)	142 000 (124 400‐154 700)	35 020 910	2 653 300 (1 970 300‐4 238 300)	100%

^a^ Estimates of persons living with HIV in each country were measured through Spectrum and published by UNAIDS and UNODC.

^b^ Median prevalence and IQRs are calculated across the best estimates for all population groups (except PWID estimates) and countries in each region (for regional estimates) or globally (for 'Total' estimates).

^c^ Proportion of HIV cases among PWID.

^d^ No regional estimate available, so global median used as a proxy.

^e^ Only one country estimate available, therefore no IQR presented.

^f^ Median prevalence and IQRs are calculated across the best PWID estimates for each country in each region (for regional estimates) or globally (for 'Total' estimates)

## 4 DISCUSSION

This is the first systematic review to provide global, regional and country estimates of prevalence and burden of HBsAg positivity among PLHIV across six population sub‐groups; complementing a companion review on HIV‐HCV antibody co‐infection.^27^ We estimate a global prevalence of 7.6% (IQR 5.6%‐12.1%) or 2.7 million (IQR 2.0‐4.2 million) cases of HIV‐HBsAg co‐infection. The greatest burden (69% of all cases; 1.9 million) is in sub‐Saharan Africa where there is the largest number of PLHIV. This is followed by 12% (355 600) in South and South‐East Asia and 6% (68 800) in Latin America.

HBsAg prevalence was broadly similar across different HIV‐positive population groups, with a prevalence of 6%‐7% reported among general population samples, heterosexually exposed or pregnant women, children, MSM and high‐risk populations. Only among PWID was prevalence higher at 11.8%. PWID accounted for only 3% of the global co‐infected population, but a much higher proportion in Eastern Europe (45% of cases). We were limited in our ability to make regional comparisons across sub‐populations because only two regions (sub‐Saharan Africa and South and South‐East Asia) had general population data, and there were little data among high‐risk populations in sub‐Saharan Africa. The most comprehensive data from different regions was among heterosexual or pregnant PLHIV. The prevalence was highest in West and Central Africa (12.0%), and East Asia (12.8%) and lowest in Latin America (2.8%) and North America (4.2%).

Our global estimate of burden of HIV‐HBsAg co‐infection is broadly consistent with previous estimates of 2‐4 million.^24^, ^25^, ^58^, ^59^ A review of HIV‐HBsAg co‐infection in sub‐Saharan Africa found a mean prevalence of 12.5% among HIV‐positive cohorts, slightly higher than we found, although that study did not disaggregate by HIV‐exposure category making comparisons challenging.^25^ Our findings broadly reflect existing data on the main routes of transmission of HBV infection. In sub‐Saharan Africa, HBV infection is predominantly acquired perinatally or in early childhood, leading to high rates of chronic infection.^4^, ^60^ The contribution of adult acquisition is low, as the majority are already chronically infected or immune. As a result, most people have already been HBV‐infected for many years by the time they are exposed to HIV in adulthood, which may explain why the prevalence is similar across different sub‐populations.^9^ In contrast, in other regions a higher prevalence occurs among PWID and MSM compared to the general population. This was particularly marked in Latin America, with co‐infection prevalence of 9.5% and 27% among PWID and MSM, respectively, compared to 0.9% in the general population. This is consistent with co‐transmission of HBV and HIV in adulthood in these settings, with much lower transmission of HBV in childhood.^22^ A significant proportion of cases in high risk populations may also represent acute infection, which will not lead to chronic infection.

This HIV‐HBsAg review and a companion review of HIV‐HCV antibody co‐infection (consistent with past or current infection)^27^ highlights important differences between the epidemiology of these co‐infections. Although the global prevalence and burden are similar (7.6% for HIV‐HBsAg compared to 6.2% for HIV‐HCV) antibody, almost three‐quarters of the global burden of HIV‐HBsAg co‐infection in 2017 is in sub‐Saharan Africa (1.91 million), 4‐5 times as many as HIV‐HCV co‐infections (429 600). In contrast, the greatest burden of HIV‐HCV co‐infection is in the concentrated epidemic settings of Central Asia and Eastern Europe among PWID, accounting for 27% of the HIV‐HCV burden (607 700). There is a much lower prevalence of HBsAg than HCV antibody among HIV‐positive PWID (11.8% vs 82%), accounting for 3% (142 000) of HIV‐HBsAg co‐infections but 59% (1.36 million) of HIV‐HCV co‐infections. Overall, we found less variability in HIV‐HBsAg prevalence between sub‐populations, and HIV infection was a less important risk factor for HBsAg positivity than for HCV antibody positivity.

Key strengths of our systematic review were the comprehensive search of published literature in English, French, Russian, Chinese, Portuguese, Arabic and Spanish; the stratification of co‐infection estimates for different population sub‐groups; and the large number of studies among heterosexually exposed and pregnant women living with HIV—the main source for regional estimates of HIV‐HBsAg co‐infection. Despite this, estimates were available for only 41% of countries, half being in sub‐Saharan Africa. Five regional prevalence estimates for different sub‐populations were based on data from a single country, possibly unrepresentative of the true regional profile. Few countries had data for all sub‐populations making regional comparisons difficult. We excluded studies that only presented regional‐level data, not disaggregated by country, to enable us to observe how prevalence varied by country and risk group within a region and to take account of the differing epidemiology of HIV at a country level. However, this resulted in the exclusion of large cohorts that aggregate across country and region. One major cohort that reports data across Europe, Israel and Argentina found a comparable prevalence of HIV‐HBsAg co‐infection (7.1%‐8.7%) with similar high prevalence observed in Argentina among PLHIV (exposure group not specified) to the range of prevalence we found among MSM.^61^, ^62^

The quality of studies was also variable. Few studies were based on large multisite surveys, with 40% of sero‐surveys being based on data from one city and fewer than 200 persons. In addition, over half of studies provided no details of the assay type and testing protocol; with much of the remainder using a recent HBsAg assay but without confirmatory HBsAg testing, possibly overestimating the infected population. In general, WHO does not recommend the use of a second confirmatory HBsAg assay for diagnosis of CHB infection (23). We also did not exclude populations based on receiving ART, with few studies reporting the prevalence of ART making it difficult to adjust for the effects of treatment. The use of tenofovir‐based ART regimens may have reduced detection of HBsAg among some samples.^63^ Finally, we have not taken into account increases in both ART coverage and the use of tenofovir‐based ART regimens that may result in lower levels of co‐infection in later years.

Our findings have important programmatic implications. First, universal infant and perinatal HBV vaccination remains the key strategy for preventing mother‐to‐child transmission and controlling the HBV epidemic. Although high uptake of infant vaccination has been achieved, leading to substantial decreases in incidence in recent years, HBV birth‐dose vaccination is being implemented by less than half of countries, and only 9/48 of countries in Africa.^4^ Rates of adult vaccination also remain low, with <3% of countries routinely vaccinating high‐risk populations (PWID, MSM, sex workers and prisoners).^22^ Our findings highlight the importance of targeting PLHIV, especially high‐risk groups and children for testing, catch‐up HBV vaccination and other preventative interventions.^64^ Second, the global scale‐up of HIV treatment for PLHIV using a tenofovir‐based ART regimen represents a major opportunity for achieving global targets towards hepatitis B elimination in PLHIV, by simultaneously treating those who have chronic and HIV co‐infection so reducing mother‐to‐child transmission of HBV alongside HIV.^65^ ART coverage is now approximately 50% in most countries and encouragingly coverage in eastern and southern Africa is higher than the global average,^66^ with 60% of persons on ART receiving a tenofovir‐based regimen.^67^ Our findings clearly show the need to scale‐up tenofovir‐based ART to address HIV‐HBsAg co‐infection, particularly focusing on sub‐Saharan Africa.

## CONFLICTS OF INTEREST

No conflicts of interest to declare.

## AUTHOR CONTRIBUTORS

PE conceived the study proposal. LP, PV and PE developed the overall methods for use in the report. LP developed the methodology and oversaw the search and data extraction for the report. CM developed and conducted the literature search. LP, CF, AT, JO, JS, BM and EG extracted data. LP, PE, HR and EG developed the quality assessment tool. LP and PV developed the analysis technique. LP and CF generated regional and global prevalence estimates, which were reviewed by PE, PV, HR and KS. KS generated the global burden of disease estimates. LP and PE led the writing of the manuscript; LP, PV and CF commented and contributed text. AT generated the maps.

## DISCLAIMER

The authors alone are responsible for the views expressed in this article and they do not necessarily represent the views, decisions or policies of the institutions with which they are affiliated, including UNAIDS and the WHO.
