# Supplementary material for: Prevalence and burden of HBV co‐infection among people living with HIV: A global systematic review and meta‐analysis
Source: J Viral Hepat. 2019 Dec 22;27(3):294–315. doi: 10.1111/jvh.13217 (PMC7383613; doi:10.1111/jvh.13217)
Supplement: Supplementary file 1 [file JVH-27-294-s001.docx]

**Supporting information**

**Supplementary material S1** Text Quality assessment process

**Text Box 1 Attributes of study design and assay quality**

| **Study design** | | **Assay type** | |
| --- | --- | --- | --- |
| A | Multi-site study with large sample (>1500 HIV cases); study design appropriate for measuring prevalence; age, sex and HIV risk categories reported | 0 | Assay type not specified |
|  |  | 1 | Detection of HBsAg Assay using a rapid test with or without a confirmatory test |
| B | >1 site study with >200 HIV cases; study design not specifically designed to measure prevalence; HIV risk categories reported | 2 | Any generation HBsAg assay **with no** confirmatory test |
| C | Single site study with <200 HIV cases; study not designed to measure prevalence; few HIV risk categories reported | 3 | Any generation HBsAg **with** confirmatory test |

**Text Box 2 Decision rules for selecting best estimates**

- If multiple co-infection estimates were available for the same population group and sites, we prioritised studies with the highest assay score, then study design score, then most recent data.
- Where more than one best estimate was identified for a given country and population group (equal highest assay score, study design score and fieldwork year), the estimate with the highest actual study design score (on a scale of 0-10) was selected.
- Co-infection estimates from one city were assumed to be from a single site unless specified otherwise.
- Where no fieldwork date was available, the year prior to publication was taken to represent timing of fieldwork assuming a lag of at least 1 year between fieldwork and publication.

**Supplementary** **Table S2: Summary of study quality based on rating of study design and assay**

| **Score** | **Total** | **%** |
| --- | --- | --- |
| A0 | 9 | 1.8 |
| A2 | 5 | 1.0 |
| A3 | 1 | 0.2 |
| B0 | 142 | 28.1 |
| B1 | 12 | 2.4 |
| B2 | 138 | 27.3 |
| B3 | 31 | 6.1 |
| C0 | 56 | 11.1 |
| C1 | 11 | 2.2 |
| C2 | 75 | 14.8 |
| C3 | 26 | 5.1 |
| Total | 506 | 100 |

**Supplementary Table S3** Summary of Global, regional and country level estimates of HIV-HBsAg co-infection prevalence estimates in children and high risk populations

*Included in separate file*

**Supplementary Table S4** Table Meta-analyses comparing HBsAg prevalence among HIV positive and negative samples among selected populations by country and region

*Included in separate file*
