# Supplementary material for: Prevalence and burden of HBV co‐infection among people living with HIV: A global systematic review and meta‐analysis
Source: J Viral Hepat. 2019 Dec 22;27(3):294–315. doi: 10.1111/jvh.13217 (PMC7383613; doi:10.1111/jvh.13217)
Supplement: Supplementary file 2 [file JVH-27-294-s002.docx]

**Supplementary Table S3:** Summary of Global, regional and country level estimates of HIV-HBsAg co-infection prevalence estimates in children and high risk populations

|  | | **Children** | | | | | | **High risk^#^** | | | | | | |
| --- | --- | --- | --- | --- | --- | --- | --- | --- | --- | --- | --- | --- | --- | --- |
|  | | **Total Studies** | | **Best estimate** | | | | **Total Studies** | | **Best estimate** | | | | |
| **Country** | | **n** | **Range^** | **%** | **Score** | **n** | **Year** | **n** | **Range^** | **%** | **Score** | | **n** | **Year** |
| **West and Central Africa** | | |  |  |  |  |  |  |  |  |  | |  |  |
| Benin^1^ | | 1 | 9.6 | 9.6 | C1 | 104 | 2014 |  |  |  |  | |  |  |
| Burkina Faso | |  |  |  |  |  |  |  |  |  |  | |  |  |
| Cameroon^2^ | |  |  |  |  |  |  | 1 | 10.6 | 10.6 | C0 | | 104 | 2013 |
| Cote D'Ivoire | |  |  |  |  |  |  |  |  |  |  | |  |  |
| Congo | |  |  |  |  |  |  |  |  |  |  | |  |  |
| Equatorial Guinea | |  |  |  |  |  |  |  |  |  |  | |  |  |
| Gambia | |  |  |  |  |  |  |  |  |  |  | |  |  |
| Ghana^3^ | |  |  |  |  |  |  | 1 | 8.7 | 8.7 | B2 | | 320 | 2012 |
| Guinea-Bissau | |  |  |  |  |  |  |  |  |  |  | |  |  |
| Mali | |  |  |  |  |  |  |  |  |  |  | |  |  |
| Nigeria^4-12^ | | 6 | 1.9-20.0 | 10.0 | C2 | 140 | 2015 | 2 | 21.2-35.7 | 21.2 | B2 | | 52 | 2013 |
| Senegal | |  |  |  |  |  |  |  |  |  |  | |  |  |
| **TOTAL^ⱡ^** | | **7** | **9.6-10.0** | **9.8** |  |  |  | **4** | **8.8-21.2** | **10.6** |  | |  |  |
| **South Africa** | |  |  |  |  |  |  |  |  |  |  | |  |  |
| Botswana | |  |  |  |  |  |  |  |  |  |  | |  |  |
| Lesotho | |  |  |  |  |  |  |  |  |  |  | |  |  |
| South Africa^13-16^ | | 4 | 0-20.5 | 0.8 | B3 | 625 | 2016 | 1 | 4.2 | 4.2 | B3 | | 1024 | 2008 |
| Zimbabwe | |  |  |  |  |  |  |  |  |  |  | |  |  |
| **TOTAL^ⱡ^** | | **4** | **0.8** | **0.8** |  |  |  | **1** | **4.2** | **4.2** |  | |  |  |
| **East Africa** | |  |  |  |  |  |  |  |  |  |  | |  |  |
| Comoros | |  |  |  |  |  |  |  |  |  |  | |  |  |
| Djibouti | |  |  |  |  |  |  |  |  |  |  | |  |  |
| Ethiopia^17^ | |  |  |  |  |  |  | 1 | 4 | 4.0 | B0 | | 305 | 2007 |
| Kenya^18^ | |  |  |  |  |  |  | 1 | 6.8 | 6.8 | B1 | | 296 | 2014 |
| Madagascar^19^ | |  |  |  |  |  |  | 1 | 6.3 | 6.3 | B1 | | 160 | 2011 |
| Malawi^20,21^ | | 1 | 2.2 | 2.2 | C2 | 91 | 2015 | 1 | 0.6 | 0.6 | B2 | | 60 | 2006 |
| Mauritius^22^ | |  |  |  |  |  |  | 1 | 0.0 | 0.0 | B2 | | 97 | 2010 |
| Mozambique | |  |  |  |  |  |  |  |  |  |  | |  |  |
| Rwanda^23^ | | 1 | 6.8 | 6.8 | C2 | 88 | 2010 |  |  |  |  | |  |  |
| Tanzania^24^ | | 1 | 7.0 | 7.0 | B2 | 157 | 2009 |  |  |  |  | |  |  |
| Uganda^25,26^ | |  |  |  |  |  |  | 2 | 7.0-19.5 | 7.0 | B2 | | 355 | 2012 |
| Zambia^27^ | | 1 | 10.5 | 10.5 | B0 | 411 | 2014 |  |  |  |  | |  |  |
| **TOTAL^ⱡ^** | | **4** | **4.5-8.7** | **6.9** |  |  |  | **7** | **0.6-6.8** | **5.1** |  | |  |  |
| **N Africa & Middle East** | |  |  |  |  |  |  |  |  |  |  | |  |  |
| Egypt^28^ | |  |  |  |  |  |  | 1 | 41.7 | 41.7 | C2 | | 115 | 2015 |
| Iran (Islamic Republic of)^29-38^ | |  |  |  |  |  |  | 10 | 3.8-35.5 | 35.5 | C3 | | 186 | 2011 |
| Lebanon^39^ | |  |  |  |  |  |  | 1 | 6.9 | 6.9 | C2 | | 101 | 2007 |
| Libya | |  |  |  |  |  |  |  |  |  |  | |  |  |
| Morocco^40^ | |  |  |  |  |  |  | 1 | 5.2 | 5.2 | B2 | | 503 | 2010 |
| Saudi Arabia^41^ | |  |  |  |  |  |  | 1 | 4.3 | 4.3 | C0 | | 1383 | 2008 |
| **TOTAL^ⱡ^** | |  |  |  |  |  |  | **14** | **5.2-3.5** | **6.9** |  | |  |  |
| **West, Central and East Europe** | | | |  |  |  |  |  |  |  |  | |  |  |
| Belgium | |  |  |  |  |  |  |  |  |  |  | |  |  |
| Bulgaria^42^ | |  |  |  |  |  |  | 1 | 10.4 | 10.4 | B2 | | 934 | 2014 |
| Denmark^43-45^ | |  |  |  |  |  |  | 3 | 2.0-7.3 | 2.0 | C2 | | 574 | 2012 |
| France^46-61^ | |  |  |  |  |  |  | 16 | 2.7-9.2 | 8.3 | B2 | | 240 | 2001 |
| Georgia^62^ | |  |  |  |  |  |  | 1 | 8.4 | 8.4 | B0 | | 752 | 2009 |
| Germany^63,64^ | |  |  |  |  |  |  | 2 | 4.5-8.2 | 4.5 | B2 | | 918 | 2010 |
| Greece | |  |  |  |  |  |  |  |  |  |  | |  |  |
| Ireland^65,66^ | |  |  |  |  |  |  | 2 | 2.1-2.7 | 2.7 | B2 | | 1287 | 2016 |
| Italy^67-91^ | |  |  |  |  |  |  | 27 | 1.0-40.0 | 8.8 | B3 | | 69 | 2013 |
| Moldova | |  |  |  |  |  |  |  |  |  |  | |  |  |
| Netherlands | |  |  |  |  |  |  |  |  |  |  | |  |  |
| Poland^92-94^ | | 1 | 2.5 | 2.5 | B3 | 79 | 2016 | 2 | 6.5-6.7 | 6.5 | C2 | | 70 | 2005 |
| Portugal | |  |  |  |  |  |  |  |  |  |  | |  |  |
| Romania^95,96^ | | 2 | 43.5-45.0 | 43.5 | C2 | 161 | 2004 |  |  |  |  | |  |  |
| Spain^97-114^ | | 1 | 1.6 | 1.6 | B0 | 127 | 2011 | 20 | 2.5-21.5 | 4.4 | B2 | | 520 | 2009 |
| Serbia^115-119^ | |  |  |  |  |  |  | 2 | 2.3-6.2 | 2.3 | B0 | | 216 | 2012 |
| Slovenia^120^ | |  |  |  |  |  |  | 1 | 3.9 | 3.9 | B2 | | 356 | 2008 |
| Switzerland^121-124^ | |  |  |  |  |  |  | 6 | 2.1-24.6 | 6.3 | C2 | | 889 | 2004 |
| Turkey^125,126^ | |  |  |  |  |  |  | 2 | 4.3-9.8 | 4.3 | B0 | | 209 | 2011 |
| United Kingdom^127-131^ | |  |  |  |  |  |  | 5 | 1.7-6.9 | 5.2 | B2 | | 2530 | 2005 |
| Ukraine^132,133^ | |  |  |  |  |  |  | 2 | 1.3-16.0 | 1.3 | B2 | | 520 | 2005 |
| **TOTAL^ⱡ^** | | **4** | **1.6-43.5** | **2.5** |  |  |  | **92** | **2.7-8.3** | **4.5** |  | |  |  |
| **East Asia** | |  |  |  |  |  |  |  |  |  |  | |  |  |
| China^134-153^ | |  |  |  |  |  |  | 20 | 1.5-19.0 | 12.0 | B3 | | 1984 | 2013 |
| Taiwan^154-157^ | |  |  |  |  |  |  | 4 | 11.5-19.8 | 19.1 | B2 | | 4273 | 2008 |
| **TOTAL^ⱡ^** | |  |  |  |  |  |  | **24** | **12.0-19.1** | **15.6** |  | |  |  |
| **South and South East Asia** | | |  |  |  |  |  |  |  |  |  | |  |  |
| Cambodia | |  |  |  |  |  |  |  |  |  |  | |  |  |
| India^158-173^ | | 1 | 2.6 | 2.6 | C0 | 487 | 2009 | 16 | 1.5-30.9 | 5.9 | B3 | | 1331 | 2013 |
| Indonesia^174-177^ | |  |  |  |  |  |  | 4 | 3.4-7.9 | 7.9 | B2 | | 126 | 2010 |
| Malaysia^178,179^ | |  |  |  |  |  |  | 2 | 9.0-13.0 | 13.0 | B0 | | 664 | 2012 |
| Myanmar^180^ | |  |  |  |  |  |  | 1 | 5.3 | 5.3 | B0 | | 11032 | 2012 |
| Nepal^181,182^ |  |  |  |  |  |  |  | 2 | 4.5-8.5 | 8.5 | B2 | | 74 | 2007 |
| Pakistan^183^ |  |  |  |  |  |  |  | 1 | 1.0 | 1.0 | B1 | | 99 | 2009 |
| Thailand^184-193^ | | 1 | 32.6 | 32.6 | B3 | 521 | 2011 | 9 | 3.7-11.4 | 11.4 | B3 | | 211 | 2013 |
| Vietnam^194,195^ |  |  |  |  |  |  |  | 2 | 10.3-11.3 | 10.3 | B2 | | 301 | 2007 |
| **TOTAL^ⱡ^** | | **2** | **2.6-32.6** | **17.6** |  |  |  | **37** | **5.6-10.8** | **8.2** |  | |  |  |
| **Asia Pacific & Australasia** | | | |  |  |  |  |  |  |  |  | |  |  |
| Australia | |  |  |  |  |  |  |  |  |  |  | |  |  |
| Japan | |  |  |  |  |  |  | 1 | 8.9 | 8.9 | B2 | | 471 | 2002 |
| South Korea^196^ | |  |  |  |  |  |  | 1 | 8.8 | 8.8 | B0 | | 327 | 2006 |
| **TOTAL^ⱡ^** | |  |  |  |  |  |  | **2** | **8.9** | **8.8-8.9** |  | |  |  |
| **Latin America (Central, South America & Caribbean)** | | | | |  |  |  |  |  |  |  | |  |  |
| Argentina^197-200^ | |  |  |  |  |  |  | 4 | 3.2-16.9 | 16.9 | C3 | | 174 | 1999 |
| Brazil^201-223^ | |  |  |  |  |  |  | 24 | 1.6-39.8 | 6.4 | B3 | | 704 | 2003 |
| Chile | |  |  |  |  |  |  |  |  |  |  | |  |  |
| Colombia^224^ | |  |  |  |  |  |  | 1 | 0.7 | 0.7 | B2 | | 251 | 2004 |
| Ecuador |  |  |  |  |  |  |  |  |  |  |  | |  |  |
| Haiti |  |  |  |  |  |  |  |  |  |  |  | |  |  |
| Mexico^225-227^ |  |  |  |  |  |  |  | 3 | 3.4-29.4 | 29.4 | B2 | | 243 | 2015 |
| Peru | |  |  |  |  |  |  |  |  |  |  | |  |  |
| Venezuela | |  |  |  |  |  |  |  |  |  |  | |  |  |
| **TOTAL^ⱡ^** | |  |  |  |  |  |  | **32** | **3.5-23.1** | **11.6** |  | |  |  |
| **North America** | |  |  |  |  |  |  |  |  |  |  | |  |  |
| Canada^228,229^ | |  |  |  |  |  |  | 2 | 6.0-19.1 | 6.0 | B3 | | 2844 | 2010 |
| USA^230-256^ | | 1 | 2.6 | 2.6 | C0 | 228 | 2005 | 28 | 1.5-56.1 | 5.9 | B2 | | 1119 | 2008 |
| **TOTAL^ⱡ^** | | **1** | **2.6** | **2.6** |  |  |  | **30** | **5.8-6.0** | **5.9** |  | |  |  |
| **Global total^ⱡ^** |  | **22** | **2.5-10.0** | **6.8** |  |  |  | **243** | **4.3-9.6** | **6.4** |  | |  |  |
| **ⱡ** Totals are derived from median of best estimates scored with interquartile range of best estimates. | | | | | | | | | | | |  |  |  |
| ^ Range is presented for country level estimates and interquartile range for regional and global totals. | | | | | | | | | | | |  |  |  |
| ^#^ Includes those recruited from STI clinics, mixed populations engaging in sexual and or injecting risk behaviours (but with less than 75% sample reporting injecting / less than 50% being MSM), sex workers and prisoners | | | | | | | | | | | |  |  |  |
| All best estimates are selected according to the decision rules in Text Box 2. | | | | | | | | | | | |  |  |  |

1. D'Almeida M, Adedemy JD, Agossou J, Noudamadjo A, Agossou C, Agossou R, Koumakpai-Adeothy S. Frequency of HIV and viral hepatitis B co-infection in children aged 1 to 15 years attended in a hospital environment in Parakou (Benin). Current Pediatric Research. 2015;19(2):81-9

2. Assob JCN, Nde PF, Nsagha DS, Njunda AL, Ngum NM, Ngowe MN. Incidence and risk factors of anti-tuberculosis drugs induced hepatotoxicity in HIV/AIDS patients attending the Limbe and Buea Regional Hospitals. Journal of AIDS and Clinical Research. 2014;5(3)

3. Kye D, Nortey P, Malm K, Nyarko KM, Sackey SO, Ofori S, Afari EA. Prevalence of hepatitis B virus co-infection among HIV-seropositive persons attending antiretroviral clinics in the Eastern Region of Ghana. The Pan African medical journal. 2016 01/01/;25(Suppl 1):7-

4. Sadoh AE, Sadoh WE, Iduoriyekemwen NJ. HIV co-infection with hepatitis B and C viruses among Nigerian children in an antiretroviral treatment programme. SAJCH South African Journal of Child Health. 2011;5(1):7-10

5. Ubesie AC, Iloh KK, Eze CU, Iloh O, Ibeziako NS, Okoli C, Emodi IJ. Clinical and Laboratory Profile of ARV Naive HIV Infected Children in the Era of Highly Active Anti-retroviral Therapy in Enugu, South-East Nigeria. Jul-2014

6. Mbaawuaga EM. Studies on prevalence, co-infection and associated risk factors of hepatitis B virus (HBV) and human immunodeficiency virus (HIV) in Benue State, Nigeria. Sexually Transmitted Diseases. 2014 June;41:S143

7. Eke AC, Eke UA, Okafor CI, Ezebialu IU, Ogbuagu C. Prevalence, correlates and pattern of hepatitis B surface antigen in a low resource setting. Virology Journal. 2011;8:12

8. Ashir GM, Rabasa AI, Gofama MM, Bukbuk D, Abubakar H, Farouk GA. Study of hepatic functions and prevalence of hepatitis B surface antigenaemia in Nigerian children with human immunodeficiency virus infection. Niger J Med. 2009 Jul-Sep;18(3):260-2

9. Davidson UN, Chidiebele NI, Josephine EI, Olakunle OE, Nnaemeka IA, Chijioke EJ, Kingsley NI. The prevalence liver function and immunologic status of children with HIV and hepatitis B virus coinfection in Enugu, Nigeria. African Journal of Infectious Diseases. 2016;10(2):61-8

10. Nwolisa E, Mbanefo F, Ezeogu J, Amadi P. Prevalence of Hepatitis B co-infection amongst HIV infected children attending a care and treatment centre in Owerri, South-eastern Nigeria. Pan African Medical Journal. 2013;14(89)

11. Jooste P, van Zyl A, Adland E, Daniels S, Hattingh L, Brits A, Wareing S, Goedhals D, Jeffery K, Andersson M, Goulder P, Matthews PC. Screening, characterisation and prevention of Hepatitis B virus (HBV) co-infection in HIV-positive children in South Africa. Journal of Clinical Virology. 2016 01;85:71-4

12. Anigilaje EA, Olutola A. Human immunodeficiency virus and hepatitis C virus co-infection among children in an antiretroviral therapy programme in Benue. Internet Journal of Infectious Diseases. 2013;12(1)

13. Barth RE, Huijgen Q, Tempelman HA, Mudrikova T, Wensing AM, Hoepelman AI. Presence of occult HBV, but near absence of active HBV and HCV infections in people infected with HIV in rural South Africa. Journal of Medical Virology. 2011 Jun;83(6):929-34

14. Beghin JC, Ruelle J, Sokal E, Bachy A, Krishna M, Hall L, Goubau P, Linden Dvd. Effectiveness of the South African expanded program of immunization against hepatitis B in children infected with human immunodeficiency virus-1 living in a resource-limited setting of KwaZulu-Natal. Journal of Medical Virology. 2017;89(1):182-5

15. Chotun N, Nel E, Cotton MF, Preiser W, Andersson MI. Hepatitis B virus infection in HIV-exposed infants in the Western Cape, South Africa. Vaccine. 2015 01/01/;33(36):4618-22

16. Mdlalose N, Parboosing R, Moodley P. The prevalence of hepatitis B virus infection in HIV-positive and HIV-negative infants: KwaZulu-Natal, South Africa. African Journal of Laboratory Medicine. 2016;5(1):a283

17. Shimelis T, Torben W, Medhin G, Tebeje M, Andualm A, Demessie F, Mulu A, Tegbaru B, Gebre-Selassie S. Hepatitis B virus infection among people attending the voluntary counselling and testing centre and anti-retroviral therapy clinic of St Paul's General Specialised Hospital, Addis Ababa, Ethiopia. Sex Transm Infect. 2008 Feb;84(1):37-41

18. Webale MK, Budambula V, Lihana R, Musumba FO, Nyamache AK, Budambula NLM, Ahmed AA, Ouma C, Were T. Hepatitis B virus sero-profiles and genotypes in HIV-1 infected and uninfected injection and Non-injection drug users from coastal Kenya.[Erratum appears in BMC Infect Dis. 2015;15:373 Note: Kilongosi, Mark W [corrected to Webale, Mark K]; PMID: 26334305]. BMC Infectious Diseases. 2015;15(299):30

19. Rakotozafindrabe ALR, Andriamifidison RN, Rabenjanahary T, Razafimahefa SH, Rakotoarivelo R, Randria MJD, Ramanampamonjy RM, Andrianasolo R. Prevalence of the coinfection by immunodeficiency human virus and hepatitis B and C viruses: Multicenter survey in Madagascar. [French]. Journal Africain d'Hepato-Gastroenterologie. 2017 01;11 (2):52-7

20. Varo R, Buck WC, Kazembe PN, Phiri S, Andrianarimanana D, Weigel R. Seroprevalence of CMV, HSV-2 and HBV among HIV-infected Malawian children: a cross-sectional survey. Journal of Tropical Pediatrics. 2016;62(3):220-6

21. Chimphambano C, Komolafe IO, Muula AS. Prevalence of HIV, HepBsAg and Hep C antibodies among inmates in Chichiri prison, Blantyre, Malawi. Malawi Medical Journal. 2007;19(3):107-10

22. Johnston LG, Corceal S. Unexpectedly high injection drug use, HIV and hepatitis C prevalence among female sex workers in the Republic of Mauritius. Aids Behav. 2013 Feb;17(2):574-84

23. Mutwa PR, Boer KR, Rusine JB, Muganga N, Tuyishimire D, Reiss P, Lange JM, Geelen SPM. Hepatitis B virus prevalence and vaccine response in HIV-infected children and adolescents on combination antiretroviral therapy in Kigali, Rwanda. Pediatric Infectious Disease Journal. 2013;32(3):246-51

24. Muro FJ, Fiorillo SP, Sakasaka P, Odhiambo C, Reddy EA, Cunningham CK, Buchanan AM. Seroprevalence of hepatitis B and C viruses among children in Kilimanjaro Region, Tanzania. Journal of the Pediatric Infectious Diseases Society. 2013 01 Dec;2(4):320-6

25. Hahn JA, Dobkin LM, Mayanja B, Emenyonu NI, Kigozi IM, Shiboski S, Bangsberg DR, Gnann H, Weinmann W, Wurst FM. Phosphatidylethanol (PEth) as a biomarker of alcohol consumption in HIV-positive patients in sub-Saharan Africa. Alcoholism: Clinical & Experimental Research. 2012 May;36(5):854-62

26. Katusiime C, Schlech WF, III, Parkes-Ratanshi R, Sempa J, Kambugu A. Characteristics of sexually transmitted infections among high-risk HIV-positive patients attending an urban clinic in Uganda. Journal of the International Association of Providers of AIDS Care. 2016;15(1):36-41

27. Peebles K, Nchimba L, Chilengi R, Moore CB, Mubiana-Mbewe M, Vinikoor MJ. Pediatric HIV-HBV coinfection in Lusaka, Zambia: prevalence and short-term treatment outcomes. Journal of Tropical Pediatrics. 2015;61(6):464-7

28. Salman TAH, Attallah KM, El-Haddad OM, Abdel-Latif AM, Awid MA. Influence of hepatitis B and hepatitis C virus infections and human immunodeficiency virus therapy on liver status of patients with human immunodeficiency virus. British Journal of Medicine and Medical Research. 2016;11(7):BJMMR-19183

29. Davarpanah MA, Fallahzadeh E, Rasti M, Rahmati H, Motazedian N. Hepatitis B virus infection serology and the associated risk factors among patients with HIV in Shiraz, Iran. Shiraz E Medical Journal. 2015;16 (4) (no pagination)(e24676)

30. Alinaghi SAS, Jam S, Mehrkhani F, Fattahi F, Sabzvari D, Kourorian Z, Jabbari H. Hepatitis-C and hepatitis-B Co-infections in patients with human immunodeficiency virus in Tehran, Iran. Acta Med Iran. 2011;49(4):252-7

31. Ramezani A, Mohraz M, Aghakhani A, Banifazl M, Eslamifar A, Khadem-Sadegh A, Velayati AA. Frequency of isolated hepatitis B core antibody in HIV-hepatitis C virus co-infected individuals. International Journal of STD & AIDS. 2009 May;20(5):336-8

32. Azadmanesh K, Mohraz M, Aghakhani A, Edalat R, Jam S. Occult hepatitis B virus infection in HIV-infected patients with isolated hepatitis B core antibody. Intervirology. 2008 2008;51(4):270-4

33. Hekmat S, Mohraz M, Vahabpour R, Jam S, Bahramali G, Banifazl M, Aghakhani A, Eslamifar A, Mahboudi F, Edalat R, Ramezani A. Frequency and genotype of GB virus C among Iranian patients infected with HIV. Journal of Medical Virology. 2008 Nov;80(11):1941-6

34. Ramezani A, Mohraz M, Banifazl M, Jam S, Gachkar L, Yaghmaie F, Eslamifar A, Zadsar M, Kalantar N, Nemati K, Haghighi M, Rezaie M, Aghakhani A. Frequency and associated factors of proteinuria in Iranian HIV-positive patients. International Journal of Infectious Diseases. 2008 Sep;12(5):490-4

35. Mohammadnejad E, Jalaimanesh S, Mahmoodi M. Clinical syndrome in HIV/AIDS resulting in hospitalization based on the CD4 count. [Persian]. Journal of Mazandaran University of Medical Sciences. 2010;20(74):69-77

36. Mohammadi M, Talei G, Sheikhian A, Ebrahimzade F, Pournia Y, Ghasemi E, Boroun H. Survey of both hepatitis B virus (HBsAg) and hepatitis C virus (HCV-Ab) coinfection among HIV positive patients. Virology Journal. 2009;6:202

37. SeyedAlinaghi S, Jam S, Mehrkhani F, Fattahi F, Sabzvari D, Kourorian Z, Jabbari H, Mohraz M. Hepatitis-C and hepatitis-B co-infections in patients with human immunodeficiency virus in Tehran, Iran. Acta Med Iran. 2011;49(4):252-7

38. Mohammad Nejad E, Ehsani SR, Rabirad N, Deljo R, Ranjbarn S, Rezaee S, Tamizi Z. Prevalence of HBV in HIV patients referred to Imam Khomeini Hospital, Tehran, Iran from 2008-2010. Iranian Red Crescent Medical Journal. 2013 April;15(4):379-80

39. Ramia S, Mokhbat J, Ramlawi F, El-Zaatari M. Occult hepatitis B virus infection in HIV-infected Lebanese patients with isolated antibodies to hepatitis B core antigen. International Journal of STD & AIDS. 2008;19(3):197-9

40. Rebbani K, Ouladlahsen A, Bensghir A, Akil A, Lamdini H, Issouf H, Brahim I, Kitab B, Fakhir FZ, Wakrim L, El Filali KM, Himmich H, Ezzikouri S, Benjelloun S. Co-infections with hepatitis B and C viruses in human immunodeficiency virus-infected patients in Morocco. Clinical Microbiology and Infection. 2013 Oct;19(10):E454-E7

41. Bajhmoum W, Beeching N, Chawla A, Geretti A, Elsyed M, Memish Z, Hopkins M. Clinical features and epidemiology of HIV and coinfection with TB and/or viral hepatitis in a large clinic in Jeddah, Kingdom of Saudi Arabia. HIV Medicine. 2014 April;15:120

42. Alexiev I, Alexandrova M, Golkocheva-Markova E, Teoharov P, Gancheva A, Kostadinova A, Dimitrova R, Elenkov I, Chervenjakova T, Stoycheva M, Nikolova D, Varleva T, Nikolova M. High rate of hepatitis B and C coinfections among people living with HIV-1 in Bulgaria: 2010-2014. AIDS Research and Human Retroviruses. 2017;33(3):228-9

43. Omland LH, Weis N, Skinhoj P, Laursen A, Christensen PB, Nielsen HI, Moller A, Engsig F, Sorensen HT, Obel N. Impact of hepatitis B virus co-infection on response to highly active antiretroviral treatment and outcome in HIV-infected individuals: a nationwide cohort study. HIV Medicine. 2008 May;9(5):300-6

44. Obel N, Reinholdt H, Omland LH, Engsig F, Sorensen HT, Hansen AB. Retrivability in The Danish National Hospital Registry of HIV and hepatitis B and C coinfection diagnoses of patients managed in HIV centers 1995-2004. BMC Med Res Methodol. 2008;8:25

45. Andersen KLD, Larsen CS, Petersen MS, Leutscher PDC. Need for improvements in the surveillance and management of chronic viral hepatitis in HIV patients followed in a Danish outpatient clinic. Scandinavian Journal of Infectious Diseases. 2014 August;46(8):578-84

46. Binquet C, le Teuff G, Abrahamovicz M, Mahboubi A, Yazdanpanah Y, Rey D, Rabaud C, Chirouze C, Berger JL, Faller JP, Chavanet P, Quantin C, Piroth L, Grp Inter CN-E. Markov modelling of HIV infection evolution in the HAART era. Epidemiology and Infection. 2009 Sep;137(9):1272-82

47. Henard S, Letranchant L, Borel A, Ajana F, Rey D, Hustache-Mathieu L, Chavanet P, May T, Rabaud C. Study of new HIV infection between 2000 and 2007 in the north and east of France. [French]

Etude des infections a VIH nouvellement decouvertes entre 2000 et 2007 dans le Nord et l'Est de la France. Medecine et Maladies Infectieuses. 2010 September;40(9):517-23

48. Larsen C, Pialoux G, Salmon D, Antona D, Piroth L, Strat YL, Pol S, Rosenthal E, Neau D, Semaille C, Delarocque-Astagneau E. Prevalence of co-infections with hepatitis B and C viruses in the HIV-positive population, France, June 2004. Bulletin Epidemiologique Hebdomadaire. 2005;23:109-12

49. Casanova M-L, Makinson A, Eymard-Duvernay S, Ouedraogo D-E, Badiou S, Reynes J, Tuaillon E. Monoclonal Gammopathy in HIV-1-Infected Patients: Factors Associated With Disappearance Under Long-Term Antiretroviral Therapy. Journal of Acquired Immune Deficiency Syndromes: JAIDS. 2015;70(3):250-5

50. de Monteynard LA, Dray-Spira R, de Truchis P, Grabar S, Launay O, Meynard JL, Khuong-Josses MA, Gilquin J, Rey D, Simon A, Pavie J, Mahamat A, Matheron S, Costagliola D, Abgrall S, French Hosp Database HIV. Later cART Initiation in Migrant Men from Sub-Saharan Africa without Advanced HIV Disease in France. Plos One. 2015 Mar;10(3)

51. Bonnet F, Pineau JJ, Taupin JL, Feyler A, Bonarek M, de Witte S, Bernard N, Lacoste D, Morlat P, Beylot J. Prevalence of cryoglobulinemia and serological markers of autoimmunity in human immunodeficiency virus infected individuals: a cross-sectional study of 97 patients. Journal of Rheumatology. 2003 Sep;30(9):2005-10

52. Neau D, Winnock M, Galperine T, Jouvencel AC, Castera L, Legrand E, Tranchant E, Balestre E, Lacoste D, Ragnaud JM, Dupon M, Lafon ME, Dabis F, Groupe d'Epidemiologie Clinique du SeA. Isolated antibodies against the core antigen of hepatitis B virus in HIV-infected patients. HIV Medicine. 2004 May;5(3):171-3

53. Fonquernie L, Dray-Spira R, Bamogo E, Lert F, Girard PM. Characteristics of newly managed HIV-infected patients: hospital Saint-Antoine, Paris 2002-2003. [French]

Caracteristiques des patients nouvellement pris en charge pour une infection VIH dans un CHU parisien en 2002-2003. Medecine et maladies infectieuses. 2006 May;36(5):270-9

54. Bruneau L, Billaud E, Raffi F, Hanf M. Factors associated with the level of CD4 cell counts at HIV diagnosis in a French cohort: a quantile regression approach. International Journal of STD & AIDS. 2017;28(4):397-403

55. de la Tribonniere X, Pugliese P, Cabie A, Cuzin L, Billaud E, Poizot-Martin I, Duvivier C, Yazdanpanah Y. Demographic, clinical, immunovirological and therapeutic features of 8714 HIV infected French patients included in the Nadis Hospital Cohorte in 2006. [French]

Caracteristiques demographiques, cliniques, immunovirologiques et therapeutiques de 8714 patients infectes par le VIH de la cohorte Nadis en 2006. Medecine et Maladies Infectieuses. 2008 June;38(6):299-308

56. Piroth L, Binquet C, Vergne M, Minello A, Livry C, Bour JB, Buisson M, Duong M, Grappin M, Portier H, Chavanet P. The evolution of hepatitis B virus serological patterns and the clinical relevance of isolated antibodies to hepatitis B core antigen in HIV infected patients. Journal of Hepatology. 2002 May;36(5):681-6

57. Bruyand M, Dabis F, Vandenhende MA, Lazaro E, Neau D, Leleux O, Geffard S, Morlat P, Chene G, Bonnet F. HIV-induced immune deficiency is associated with a higher risk of hepatocarcinoma, ANRS CO3 Aquitaine Cohort, France, 1998-2008. Journal of Hepatology. 2011 Nov;55(5):1058-62

58. Hentzien M, Drame M, Allavena C, Jacomet C, Valantin MA, Cabie A, Cuzin L, Rey D, Pugliese P, Bani-Sadr F. Impact of age-related comorbidities on five-year overall mortality among elderly HIV-infected patients in the late HAART era - role of chronic renal disease. Journal of Nutrition, Health & Aging. 2016;20(4):408-14

59. Mary-Krause M, Grabar S, Lievre L, Abgrall S, Billaud E, Boue F, Boyer L, Cabie A, Cotte L, De Truchis P, Duval X, Duvivier C. Cohort Profile: French hospital database on HIV (FHDH-ANRS CO4). International Journal of Epidemiology. 2014 Oct;43(5):1425-36

60. Flexor G, Zucman D, Berthe H, Meier F, Force G, Greder-Belan A, Billy C, Dupont C, Mortier E, Bizard A, Rouveix E, De Truchis P. Aging and HIV infection: 4years follow-up of 149 HIV infected patients older than 60years in West Paris agglomeration (COREVIH* ile-de-France Ouest). [French]. Presse Medicale. 2013 May;42(5):e145-e52

61. Duval X, Journot V, Leport C, Chene G, Dupon M, Cuzin L, May T, Morlat P, Waldner A, Salamon R, Raffi F. Incidence of and risk factors for adverse drug reactions in a prospective cohort of HIV-infected adults initiating protease inhibitor-containing therapy. Clinical infectious diseases : an official publication of the Infectious Diseases Society of America. 2004 Jul 15;39(2):248-55

62. Tsertsvadze T, Chkhartishvili N, Sharvadze L, Dvali N, Chokoshvili O, Gabunia P, Abutidze A, Nelson K, DeHovitz J, Rio Cd. Outcomes of universal access to antiretroviral therapy (ART) in Georgia. AIDS Research and Treatment. 2011;621078(31)

63. Bickel M, Marben W, Betz C, Khaykin P, Stephan C, Gute P, Haberl A, Knecht G, Wolf T, Brodt H, Geiger H, Herrmann E, Jung O. End-stage renal disease and dialysis in HIV-positive patients: Observations from a long-term cohort study with a follow-up of 22 years. HIV Medicine. 2013 March;14(3):127-35

64. Reuter S, Oette M, Wilhelm FC, Beggel B, Kaiser R, Balduin M, Schweitzer F, Verheyen J, Adams O, Lengauer T, Fatkenheuer G, Pfister H, Haussinger D. Prevalence and characteristics of hepatitis B and C virus infections in treatment-naive HIV-infected patients. Medical Microbiology and Immunology. 2011 February;200(1):39-49

65. Sadlier C, O'Rourke A, Carr A, Bergin C. Seroepidemiology of hepatitis A, hepatitis B and varicella virus in people living with HIV in Ireland. Journal of Infection and Public Health. 2017;10(6):888-90

66. O'Connell S, Lillis D, Cotter A, O'Dea S, Tuite H, Fleming C, Crowley B, Fitzgerald I, Dalby L, Barry H, Shields D, Norris S, Plunkett PK, Bergin C. Opt-out panel testing for HIV, hepatitis B and hepatitis C in an urban emergency department: a pilot study. PLoS ONE. 2016;11(3):e0150546

67. Aceti A, Pasquazzi C, Zechini B. Alanine aminotransferase decrease in HIV-hepatitis C virus co-infected patients responding to antiretroviral therapy. Aids. 2003;17(14):2141-2

68. Tozzi V, Balestra P, Lorenzini P, Bellagamba R, Galgani S, Corpolongo A, Vlassi C, Larussa D, Zaccarelli M, Noto P, Visco-Comandini U, Giulianelli M, Ippolito G, Antinori A, Narciso P. Prevalence and risk factors for human immunodeficiency virus-associated neurocognitive impairment, 1996 to 2002: results from an urban observational cohort. Journal of Neurovirology. 2005 Jul;11(3):265-73

69. Rosso R, Rossotti R, Di Biagio A, Nicolini L, Adorni F, Orani A, Viscoli C. Nevirapine-based regimens in routine clinical settings: results from a large Italian cohort of HIV-1 infected adults. Curr Drug Saf. 2011 Jul;6(3):138-44

70. Floridia M, Masuelli G, Tamburrini E, Spinillo A, Simonazzi G, Guaraldi G, Degli Antoni AM, Martinelli P, Portelli V, Dalzero S, Ravizza M. HBV coinfection is associated with reduced CD4 response to antiretroviral treatment in pregnancy. Hiv Clinical Trials. 2017 Mar;18(2):54-9

71. Fabris P, Tositti G, Giordani MT, Romano L, Betterle C, Pignattari E, Tagliaferri C, Muratori P, Manfrin V, Lalla Fd. Prevalence and clinical significance of circulating cryoglobulins in HIV-positive patients with and without coinfection with hepatitis C virus. Journal of Medical Virology. 2003;69(3):339-43

72. Foca E, Magro P, Motta D, Compostella S, Casari S, Bonito A, Brianese N, Ferraresi A, Rodari P, Pezzoli MC, Quiros-Roldan E, Castelli F. Screening for neurocognitive impairment in HIV-infected individuals at first contact after HIV diagnosis: the experience of a large clinical center in northern Italy. International Journal of Molecular Sciences. 2016;17(4):434

73. Pontali E, Bobbio N, Zaccardi M, Urciuoli R. Blood-borne viral co-infections among human immunodeficiency virus-infected inmates. International Journal of Prisoner Health. 2016;12(2):88-97

74. Cicconi P, Cozzi-lepri A, Orlando G, Matteelli A, Girardi E, Degli Esposti A, Moioli C, Rizzardini G, Chiodera A, Ballardini G, Tincati C, d'Arminio Monforte A, Group ICNAS. Recent acquired STD and the use of HAART in the Italian Cohort of Naive for Antiretrovirals (I.Co.N.A): analysis of the incidence of newly acquired hepatitis B infection and syphilis. Infection. 2008 Feb;36(1):46-53

75. Torti C, Costarelli S, De Silvestri A, Quiros-Roldan E, Lapadula G, Cologni G, Paraninfo G, Castelnuovo F, Puoti M, Carosi G, Group BS. Analysis of severe hepatic events associated with nevirapine-containing regimens: CD4+ T-cell count and gender in hepatitis C seropositive and seronegative patients. Drug Saf. 2007;30(12):1161-9

76. Sanarico N, D'Amato S, Bruni R, Rovetto C, Salvi E, Di Zeo P, Chionne P, Madonna E, Pisani G, Costantino A, Equestre M, Tosti ME, Cenci A, Maggiorella MT, Sernicola L, Pontali E, Pansera A, Quattrocchi R, Carbonara S, Signorile F. Correlates of infection and molecular characterization of blood-borne HIV, HCV, and HBV infections in HIV-1 infected inmates in Italy: An observational cross-sectional study. Medicine. 2016;95(44):e5257-e

77. Filippini P, Coppola N, Scolastico C, Rossi G, Battaglia M, Onofrio M, Pisapia R, Marrocco C, Sagnelli C, Piccinino F, Sagnelli E. Hepatitis viruses and HIV infection in the Naples area. Infezioni in Medicina. 2003 Sep;11(3):139-45

78. Cicconi P, Cozzi-Lepri A, Phillips A, Puoti M, Antonucci G, Manconi PE, Tositti G, Colangeli V, Lichtner M, Monforte A, Group ICS. Is the increased risk of liver enzyme elevation in patients co-infected with HIV and hepatitis virus greater in those taking antiretroviral therapy? AIDS. 2007 Mar 12;21(5):599-606

79. Puoti M, Torti C, Ripamonti D, Castelli F, Zaltron S, Zanini B, Spinetti A, Putzolu V, Casari S, Tomasoni L, Quiros-Roldan E, Favret M, Berchich L, Grigolato P, Callea F, Carosi G, Group H-HC-IS. Severe hepatotoxicity during combination antiretroviral treatment: incidence, liver histology, and outcome. Journal of Acquired Immune Deficiency Syndromes: JAIDS. 2003 Mar 1;32(3):259-67

80. Madeddu G, Bonfanti P, De Socio GV, Carradori S, Grosso C, Marconi P, Penco G, Rosella E, Miccolis S, Melzi S, Mura MS, Landonio S, Ricci E, Quirino T, Group C. Tenofovir renal safety in HIV-infected patients: results from the SCOLTA Project. Biomedicine & Pharmacotherapy. 2008 Jan;62(1):6-11

81. Martinelli C, Corsi P, Mena M, Carocci A. Long-term efficacy and safety of treatment with nevirapine plus nucleoside reverse transcriptase inhibitors for HIV-1 infection: An eight-years follow-up. HIV and AIDS Review. 2012 September;11(3):61-4

82. Castagna A, Galli L, Torti C, D'Arminio Monforte A, Mussini C, Antinori A, Cozzi-Lepri A, Ladisa N, De Luca A, Seminari E, Gianotti N, Lazzarin A. Predicting the magnitude of short-term CD4+ T-cell recovery in HIV-infected patients during first-line highly active antiretroviral therapy. Antiviral Therapy. 2010;15(2):165-75

83. Ripamonti D, Arici C, Pezzotti P, Maggiolo F, Ravasio L, Suter F. Hepatitis C infection increases the risk of the modification of first highly active antiretroviral therapy in HIV-infected patients. Aids. 2004 Jan;18(2):334-7

84. Menzaghi B, Ricci E, Carenzi L, Parruti G, Orofino G, Guastavigna M, Madeddu G, Maggi P, Biagio Ad, Corsi P, Penco G, Socio Gd, Martinelli C, Vichi F, Celesia BM, Franzetti M, Grosso C, Molteni C, Bonfanti P, Quirino T. Safety and durability in a cohort of HIV-1 positive patients treated with once and twice daily darunavir-based therapy (SCOLTA Project). Biomedicine & Pharmacotherapy. 2013;67(4):293-8

85. Meraviglia P, Schiavini M, Castagna A, Vigano P, Bini T, Landonio S, Danise A, Moioli MC, Angeli E, Bongiovanni M, Hasson H, Duca P, Cargnel A. Lopinavir/ritonavir treatment in HIV antiretroviral-experienced patients: evaluation of risk factors for liver enzyme elevation. HIV Medicine. 2004 Sep;5(5):334-43

86. Rossetti B, Bai F, Tavelli A, Galli M, Antinori A, Castelli F, Pellizzer G, Cozzi-Lepri A, Bonora S, Monforte ADA, Puoti M, De Luca A. Evolution of the prevalence of hepatitis C virus infection and hepatitis C virus genotype distribution in human immunodeficiency virus-infected patients in Italy between 1997 and 2015. Clinical Microbiology and Infection. 2018 April;24 (4):422-7

87. Monarca R, Madeddu G, Ranieri R, Carbonara S, Leo G, Sardo M, Choroma F, Casari S, Marri D, Muredda AA, Nava FA, Babudieri S. HIV treatment and care among Italian inmates: a one-month point survey. BMC Infectious Diseases. 2015;15(1):562-

88. De Luca A, Bugarini R, Lepri AC, Puoti M, Girardi E, Antinori A, Poggio A, Pagano G, Tositti G, Cadeo G, Macor A, Toti M, D'Arminio Monforte A, Italian Cohort Naive Antiretrovirals Study G. Coinfection with hepatitis viruses and outcome of initial antiretroviral regimens in previously naive HIV-infected subjects. Arch Intern Med. 2002 Oct 14;162(18):2125-32

89. Ravasi G, Lauriola M, Tinelli C, Brandolini M, Uglietti A, Maserati R. Comparison of glomerular filtration rate estimates vs. 24-h creatinine clearance in HIV-positive patients. HIV Medicine. 2009 Apr;10(4):219-28

90. Madeddu G, Spanu A, Chessa F, Calia GM, Lovigu C, Solinas P, Mannazzu M, Falchi A, Mura MS, Madeddu G. Thyroid function in human immunodeficiency virus patients treated with highly active antiretroviral therapy (HAART): a longitudinal study. Clin Endocrinol (Oxf). 2006 Apr;64(4):375-83

91. Torti C, Quiros-Roldan E, Tirelli V, Regazzi-Bonora M, Moretti F, Pierotti P, Orani A, Maggi P, Cargnel A, Patroni A, De Luca A, Carosi G, Cohort RSGotM. Lopinavir plasma levels in salvage regimes by a population of highly active antiretroviral therapy-treated HIV-1-positive patients. AIDS Patient Care STDS. 2004 Nov;18(11):629-34

92. Leszczyszyn-Pynka M, Wnuk A, Bander D, Boron-Kaczmarska A. Immune restoration disease in HIV infected patients during first 6 months of highly active antiretroviral therapy (HAART). HIV and AIDS Review. 2005 May;4(2):19-23

93. Pokorska-Spiewak M, Stanska-Perka A, Popielska J, Oldakowska A, Coupland U, Zawadka K, Szczepanska-Putz M, Marczynska M. Prevalence and predictors of liver disease in HIV-infected children and adolescents. Scientific Reports. 2017 Sep;7

94. Jablonowska E, Malolepsza E. Causes of death in HIV-infected patients in the region of Lodz, Poland from 1995 through 2005. Central European Journal of Medicine. 2009 June;4(2):179-83

95. Arbune M, Benea OE. Particularities of HBV-HIV co-infection in the youth from galafi. Journal of Gastrointestinal and Liver Diseases. 2012 October;21:56

96. Ruta SM, Matusa RF, Sultana C, Manolescu L, Kozinetz CA, Kline MW, Cernescu C. High prevalence of hepatitis B virus markers in Romanian adolescents with human immunodeficiency virus infection. MedGenMed. 2005;7(1):68

97. Dapena M, Jimenez B, Noguera-Julian A, Soler-Palacin P, Fortuny C, Lahoz R, Aracil FJ, Figueras C, de Jose MI. Metabolic disorders in vertically HIV-infected children: future adults at risk for cardiovascular disease. J Pediatr Endocrinol. 2012;25(5-6):529-35

98. Rivas P, Herrero MD, Poveda E, Madejon A, Trevino A, Gutierrez M, de Guevara CL, Lago M, de Mendoza C, Soriano V, Puente S. Hepatitis B, C, and D and HIV Infections among Immigrants from Equatorial Guinea Living in Spain. American Journal of Tropical Medicine and Hygiene. 2013 Apr;88(4):789-94

99. Estrada V, Geijo P, Fuentes-Ferrer M, Alcalde ML, Rodrigo M, Galindo MJ, Munoz A, Domingo P, Ribera E, Cosin J, Viciana P, Lozano F, Terron A, Vergara A, Teira R, Munoz-Sanchez J, Roca B, Sanchez T, Lopez-Aldeguer J, Deig E, Vidal F, Pedrol E, Castano-Carracedo M, Puig T, Garrido M, Suarez-Lozano I. Dyslipidaemia in HIV-infected women on antiretroviral therapy. Analysis of 922 patients from the Spanish VACH cohort. BMC Womens Health. 2011;11:36

100. Navarro-Mercade J, Crespo M, Falco V, van den Eynde E, Curran A, Burgos J, del Saz SV, Caballero E, Ocana I, Perez-Bernal M, Ribera E, Pahissa A. Long-term effectiveness of first-line antiretroviral theraphy in a cohort of HIV-1 infected patients. Journal of Antivirals and Antiretrovirals. 2012;4(2):26-31

101. Jaen A, Esteve A, Miro JM, Tural C, Montoliu A, Ferrer E, Riera M, Segura F, Force L, Sued O, Vilaro J, Garcia I, Masabeu A, Altes J, Clotet B, Podzamczer D, Murillas J, Navarro G, Gatell JM, Casabona J, Piscis Study G. Determinants of HIV progression and assessment of the optimal time to initiate highly active antiretroviral therapy - PISCIS cohort (Spain). Jaids-Journal of Acquired Immune Deficiency Syndromes. 2008 Feb;47(2):212-20

102. Blanco F, Barreiro P, Ryan P, Vispo E, Martin-Carbonero L, Tuma P, Labarga P, Medrano J, Gonzalez-Lahoz J, Soriano V. Risk factors for advanced liver fibrosis in HIV-infected individuals: role of antiretroviral drugs and insulin resistance. Journal of Viral Hepatitis. 2011 Jan;18(1):11-6

103. Gonzalez-Garcia JJ, Mahillo B, Hernandez S, Pacheco R, Diz S, Garcia P, Esteban H, Arribas JR, Quereda C, Rubio R, Diez J, Moreno S, Vazquez-Rodriguez JJ. [Prevalences of hepatitis virus coinfection and indications for chronic hepatitis C virus treatment and liver transplantation in Spanish HIV-infected patients. The GESIDA 29/02 and FIPSE 12185/01 Multicenter Study]. Enfermedades Infecciosas y Microbiologia Clinica. 2005 Jun-Jul;23(6):340-8

104. Vispo E, Fernandez-Montero JV, Labarga P, Barreiro P, Soriano V. Liver toxicity of the most recently approved antiretroviral drugs in HIV-infected patients - Overall low rate but increased in the presence of chronic hepatitis C. Journal of Hepatology. 2012 April;56:S540-S1

105. Macias J, Orihuela F, Rivero A, Viciana P, Marquez M, Portilla J, Rios MJ, Munoz L, Pasquau J, Castano MA, Abdel-Kader L, Pineda JA, Hepatip Study G. Hepatic safety of tipranavir plus ritonavir (TPV/r)-based antiretroviral combinations: effect of hepatitis virus co-infection and pre-existing fibrosis. Journal of Antimicrobial Chemotherapy. 2009 Jan;63(1):178-83

106. Cifuentes C, Mira JA, Vargas J, Neukam K, Escassi C, Garcia-Rey S, Gilabert I, Gonzalez-Monclova M, Bernal S, Pineda JA. Prevalence of hepatitis virus infection markers in HIV-infected patients in Southern Spain. [Spanish]

Prevalencia de los marcadores de infeccion de los virus de las hepatitis en pacientes portadores del VIH en el sur de Espana. Enfermedades Infecciosas y Microbiologia Clinica. 2012 October;30(8):452-7

107. Perez Cachafeiro S, Caro-Murillo AM, Berenguer J, Segura F, Gutierrez F, Vidal F, Martinez-Perez MA, Sola J, Muga R, Moreno S, Cohort of the Spanish Aids Research N. Association of Patients' Geographic Origins with Viral Hepatitis Co-infection Patterns, Spain. Emerging Infectious Diseases. 2011 Jun;17(6):1116-9

108. Lopez Calvo S, Vela A, Castro A, Cid A, Aguilera A, Vega P, Hermida M, Regueiro BJ, Pedreira JD. GB virus C: Lack of association with aminotransferase levels, CD4 and HIV viral load in aids patients. [Spanish]

Virus GB-C: Ausencia de asociacion con niveles de transaminasas, CD4 y carga virica en pacientes con sida. Anales de Medicina Interna. 2003 01 Apr;20(4):175-8

109. Pons I, Sanfeliu I, Nogueras MM, Sala M, Cervantes M, Amengual MJ, Segura F. Seroprevalence of Bartonella spp. infection in HIV patients in Catalonia, Spain. BMC Infectious Diseases. 2008;8:58

110. Castellares C, Barreiro P, Martin-Carbonero L, Labarga P, Vispo ME, Casado R, Galindo L, Garcia-Gasco P, Garcia-Samaniego J, Soriano V. Liver cirrhosis in HIV-infected patients: prevalence, aetiology and clinical outcome. Journal of Viral Hepatitis. 2008 Mar;15(3):165-72

111. Crespo M, Navarro J, Moreno S, Sanz J, Marquez M, Zamora J, Ocampo A, Iribaren JA, Rivero A, Llibre JM. Hepatic safety of maraviroc in HIV-1-infected patients with hepatitis C and/or B co-infection. The maraviroc cohort Spanish group. Enfermedades Infecciosas y Microbiologia Clinica. 2017;35(8):493-8

112. Ena J, Amador C, Benito C, Fenoll V, Pasquau F. Risk and determinants of developing severe liver toxicity during therapy with nevirapine-and efavirenz-containing regimens in HIV-infected patients. International Journal of STD & AIDS. 2003 Nov;14(11):776-81

113. Martin-Carbonero L, Nunez M, Gonzalez-Lahoz J, Soriano V. Incidence of liver injury after beginning antiretroviral therapy with efavirenz or nevirapine. HIV Clinical Trials. 2003 Mar-Apr;4(2):115-20

114. Podzamczer D, Olmo M, Sanz J, Boix V, Negredo E, Knobel H, Domingo P, Pineda JA, Vilades C, Quero JH, Force L, Lahoz JG, Munoz P, Llibre JM, Marino A, Ortega E, Dalmau D, Gatell JM, Anton E, Sola J, Galindo MJ, Pedrol E, Sanz J, Lima JT, Flores J, Group NOS. Safety of Switching Nevirapine Twice Daily to Nevirapine Once Daily in Virologically Suppressed Patients. Journal of Acquired Immune Deficiency Syndromes: JAIDS. 2009 Apr 1;50(4):390-6

115. Dragovic G, Salemovic D, Ranin J, Nikolic J, Kusic J, Jevtovic D. Clinical and immunologic outcomes of HAART-treated HIV-infected women in resource constrain settings: the Belgrade Study. Women Health. 2014;54(1):35-47

116. Jevtovic D, Dragovic G, Salemovic D, Ranin J, Kusic J, Marinkovic J, Djurkovic-Djakovic O. Treatment outcome of HAART-treated patients in a resource-limited setting: the Belgrade Cohort Study. Biomedicine & Pharmacotherapy. 2014 Apr;68(3):391-5

117. Fernandez-Montero JV, Vispo E, Barreiro P, De Mendoza C, Triano C, Cornelli B, Soriano V. Hepatitis C coinfection independently increases cardiovascular risk in HIV-infected patients. Journal of Hepatology. 2014 April;1):S322

118. Sprenger K, Evison JM, Zwahlen M, Vogt C, Elzi MV, Hauser C, Furrer H, Low N, Hubert V, Barth J, Battegay M, Bernasconi E, Boni J, Bucher HC, Burton-Jeangros C, Calmy A, Cavassini M, Egger M, Elzi L, Fehr J, Fellay J, Fux CA, Gorgievski M, Gunthard H, Haerry D, Hasse B, Hirsch HH, Hosli I, Kahlert C, Kaiser L, Keiser O, Klimkait T, Kouyos R, Kovari H, Ledergerber B, Martinetti G, de Tejada MB, Metzner K, Muller N, Nadal D, Pantaleo G, Rauch A, Regenass S, Rickenbach M, Rudin C, Schoni-Affolter F, Schmid P, Schultze D, Schupbach J, Speck R, Staehelin C, Tarr P, Telenti A, Trkola A, Vernazza P, Weber R, Yerly S. Sexually transmitted infections in HIV-infected people in Switzerland: Cross-sectional study. PeerJ. 2014;2014(1)

119. Wandeler G, Gsponer T, Bihl F, Aubert V, Barth J, Battegay M, Bernasconi E, Boni J, Bucher HC, Burton-Jeangros C, Calmy A, Cavassini M, Egger M, Elzi L, Fehr J, Fellay J, Francioli P, Furrer H, Fux CA, Gorgievski M, Gunthard H, Haerry D, Hasse B, Hirsch HH, Hirschel B, Hosli I, Kahlert C, Kaiser L, Keiser O, Kind C, Klimkait T, Kovari H, Ledergerber B, Martinetti G, Martinez De Tejada B, Metzner K, Muller N, Nadal D, Pantaleo G, Rauch A, Regenass S, Rickenbach M, Rudin C, Schmid P, Schultze D, Schoni-Affolter F, Schupbach J, Speck R, Taffe P, Tarr P, Telenti A, Trkola A, Vernazza P, Weber R, Yerly S. Hepatitis B virus infection is associated with impaired immunological recovery during antiretroviral therapy in the Swiss HIV cohort study. Journal of Infectious Diseases. 2013 01 Nov;208(9):1454-8

120. Seme K, Lunar MM, Tomazic J, Vidmar L, Karner P, Maticic M, Poljak M. Low prevalence of hepatitis B and C infections among HIV-infected individuals in Slovenia: a nation-wide study, 1986-2008. Acta dermatovenerolog. 2009 Dec;18(4):153-6

121. Marzolini C, Elzi L, Gibbons S, Weber R, Fux C, Furrer H, Chave JP, Cavassini M, Bernasconi E, Calmy A, Vernazza P, Khoo S, Ledergerber B, Back D, Battegay M, Swiss HIVCS. Prevalence of comedications and effect of potential drug-drug interactions in the Swiss HIV Cohort Study. Antiviral Therapy. 2010;15(3):413-23

122. Kaufmann GR, Elzi L, Weber R, Furrer H, Giulieri S, Vernazza P, Bernasconi E, Hirschel B, Battegay M, Swiss HIVCS. Interruptions of cART limits CD4 T-cell recovery and increases the risk for opportunistic complications and death. AIDS. 2011 Feb 20;25(4):441-51

123. Conen A, Fehr J, Glass TR, Furrer H, Weber R, Vernazza P, Hirschel B, Cavassini M, Bernasconi E, Bucher HC, Battegay M, Swiss HIVCS. Self-reported alcohol consumption and its association with adherence and outcome of antiretroviral therapy in the Swiss HIV Cohort Study. Antiviral Therapy. 2009;14(3):349-57

124. Franceschi S, Polesel J, Rickenbach M, Dal Maso L, Probst-Hensch NM, Fux C, Cavassini M, Hasse B, Kofler A, Ledergerber B, Erb P, Clifford GM. Hepatitis C virus and non-Hodgkin's lymphoma: Findings from the Swiss HIV Cohort Study. Br J Cancer. 2006 Dec 4;95(11):1598-602

125. Kaptan F, Ormen B, Turker N, El S, Ural S, Vardar I, Coskun NA, Er H, Unal Z. Retrospective evaluation of 128 cases infected with human immunodeficiency virus

Insan immun yetmezlik virusu ile enfekte 128 olgunun retrospektif olarak degerlendirilmesi. Turkiye Klinikleri Journal of Medical Sciences. 2011;31(3):525-33

126. Karaosmanoglu H, Aydin O, Nazlican O. Isolated anti-HBc among HIV-infected patients in Istanbul, Turkey. HIV Clinical Trials. 2013 01 Jan;14(1):17-20

127. Price H, Bansi L, Sabin CA, Bhagani S, Burroughs A, Chadwick D, Dunn D, Fisher M, Main J, Nelson M, Pillay D, Rodger A, Taylor C, Gilson R. Hepatitis B Virus Infection in HIV-Positive Individuals in the UK Collaborative HIV Cohort (UK CHIC) Study. PLoS ONE. 2012 07 Nov;7(11)

128. Natarajan U, Pym A, McDonald C, Velisetty P, Edwards SG, Hay P, Welch J, de Ruiter A, Taylor GP, Anderson J. Safety of nevirapine in pregnancy. HIV Medicine. 2007 Jan;8(1):64-9

129. Hakeem L, Thomson G, McCleary E, Bhattacharyya D, Banerjee I. Prevalence and Immunization Status of Hepatitis B Virus in the HIV Cohort in Fife, Scotland. Journal of clinical medicine research. 2010 01/01/

130. Ibrahim F, Naftalin C, Cheserem E, Roe J, Campbell LJ, Bansi L, Hendry BM, Sabin C, Post FA. Immunodeficiency and renal impairment are risk factors for HIV-associated acute renal failure. AIDS. 2010 Sep 10;24(14):2239-44

131. Nebbia G, Garcia-Diaz A, Ayliffe U, Smith C, Dervisevic S, Johnson M, Gilson R, Tedder R, Geretti AM. Predictors and kinetics of occult hepatitis B virus infection in HIV-infected persons. Journal of Medical Virology. 2007 Oct;79(10):1464-71

132. Landes M, Newell ML, Barlow P, Fiore S, Malyuta R, Martinelli P, Posokhova S, Savasi V, Semenenko I, Stelmah A, Tibaldi C, Thorne C. Hepatitis B or hepatitis C coinfection in HIV-infected pregnant women in Europe. HIV Medicine. 2008 Aug;9(7):526-34

133. Bailey H, Nizova N, Martsynovska V, Volokha A, Malyuta R, Cortina-Borja M, Thorne C, Pilipenko T, Posokhova S, Kaleeva T, Barishnikova Y, Servetsky S, Tereshenko R, Solokha S, Grazhdanov MP, Kulakovskaya E, Raus I, Yurchenko OV, Adejnova I, Ruban Z, Govorun O, Kochergina I, Ostrovskaya L, Primak N, Kvasha L, Kruglenko G. HCV co-infection and markers of liver injury and fibrosis among HIV-positive childbearing women in Ukraine: Results from a cohort study. BMC Infectious Diseases. 2016;16 (1) (no pagination)(755)

134. Zhao Y, Su S, Lv C, Zhang X, Lin L, Sun X, Lin B, Fu J. Seroprevalence of hepatitis C, hepatitis B virus and syphilis in HIV-1 infected patients in Shandong, China. International Journal of STD & AIDS. 2012;23(9):639-43

135. He N, Chen L, Lin HJ, Zhang M, Wei J, Yang JH, Gabrio J, Rui BL, Zhang ZF, Fu ZH, Ding YY, Zhao GM, Jiang QW, Detels R. Multiple viral coinfections among HIV/AIDS patients in China. Bioscience Trends. 2011;5(1):1-9

136. Xie J, Han Y, Qiu Z, Li Y, Li Y, Song X, Wang H, Thio CL, Li T. Prevalence of hepatitis B and C viruses in HIV-positive patients in China: a cross-sectional study. Journal of the International AIDS Society. 2016;19(1)

137. Zhang F, Zhou S, Chen X, Ji G, Lu Y, Wu Y, Liu Z, Shang H. Seroprevalence of HBV and HCV among HIV-infected adults in China according to route of HIV infection: High rate of HBV and HCV exposure. Future Virology. 2012 October;7(10):1015-20

138. Zhang T, He N, Ding Y, Crabtree K, Minhas V, Wood C. Prevalence of human herpesvirus 8 and hepatitis C virus in a rural community with a high risk for blood-borne infections in central China. Clinical Microbiology & Infection. 2011 Mar;17(3):395-401

139. Shen Y, Wang Z, Qi T, Jiang X, Song W, Tang Y, Wang J, Liu L, Zhang R, Zheng Y, Dai Z, Lu H. Serological survey of viral hepatitis markers among newly diagnosed patients with HIV/AIDS in China. HIV Medicine. 2013 March;14(3):167-75

140. Su S, Fairley CK, Sasadeusz J, He J, Wei X, Zeng H, Jing J, Mao L, Chen X, Zhang L. HBV, HCV, and HBV/HCV co-infection among HIV-positive patients in Hunan province, China: Regimen selection, hepatotoxicity, and antiretroviral therapy outcome. Journal of Medical Virology. 2018 March;90 (3):518-25

141. Ding Y, Duan S, Ye R, Yang Y, Yao S, Wang J, Cao D, Liu X, Lu L, Jia M, Wu Z, He N. More improvement than progression of liver fibrosis following antiretroviral therapy in a longitudinal cohort of HIV-infected patients with or without HBV and HCV co-infections. Journal of Viral Hepatitis. 2017;24(5):412-20

142. Yijia L, Jing X, Yang H, Huanling W, Wei L, Fuping G, Zhifeng Q, Yanling L, Shanshan D, Xiaojing S, Ting Z, Thio CL, Taisheng L, Li Y, Xie J, Han Y, Wang H, Lv W, Guo F, Qiu Z. Combination Antiretroviral Therapy Is Associated With Reduction in Liver Fibrosis Scores in HIV-1-Infected Subjects. Medicine. 2016;95(5):1-7

143. Zeng QL, Yu ZJ. Prevalence of Hepatitis C Virus and Hepatitis B Virus Infections in Patients Infected with HIV Through Blood Donation or Transfusion in China. AIDS Research and Human Retroviruses. 2015 01;31 (12):1203

144. Wei Q, Lin H, Ding Y, Liu X, Wu Q, Shen W, Gao M, He N. Liver fibrosis after antiretroviral therapy in a longitudinal cohort of sexually infected HIV patients in eastern China. Bioscience trends. 2017 24;11 (3):274-81

145. Spillane H, Nicholas S, Tang Z, Szumilin E, Balkan S, Pujades-Rodriguez M. Incidence, risk factors and causes of death in an HIV care programme with a large proportion of injecting drug users. Tropical Medicine and International Health. 2012 October;17(10):1255-63

146. Wu SL, Yan PP, Yang TF, Wang ZH, Yan YS. Epidemiological Profile and Risk Factors of HIV and HBV/HCV Co-Infection in Fujian Province, Southeastern China. Journal of Medical Virology. 2017 Mar;89(3):443-9

147. Mo P, Zhu Q, Teter C, Yang R, Deng L, Yan Y, Chen J, Zeng J, Gui X. Prevalence, drug-induced hepatotoxicity, and mortality among patients multi-infected with HIV, tuberculosis, and hepatitis virus. International Journal of Infectious Diseases. 2014;28:95-100

148. Chen X, He J, Ding L, Zhang G, Zou X, Zheng J. Prevalence of hepatitis B virus and hepatitis C virus in patients with human immunodeficiency virus infection in central China. Archives of Virology. 2013;158(9):1889-94

149. Zhang F, Zhu H, Wu Y, Dou Z, Zhang Y, Kleinman N, Bulterys M, Wu Z, Ma Y, Zhao D, Liu X, Fang H, Liu J, Cai W, Shang H. HIV, hepatitis B virus, and hepatitis C virus co-infection in patients in the China National Free Antiretroviral Treatment Program, 2010-12: a retrospective observational cohort study. Lancet Infectious Diseases. 2014;14(11):1065-72

150. Chen J-J, Yu C-B, Du W-B, Li L-J. Prevalence of hepatitis B and C in HIV-infected patients: a meta-analysis. Hepatobiliary Pancreat Dis Int. 2011 Apr;10(2):122-7

151. Ding L, Zhang G, Jiang Y, Qin B, He J, Chen X, Zou X, Zhang F. Infection status and risk factors of HBV and HCV among HIV-infected people in Hunan, China. Chinese Journal of Viral Diseases. 2011;1(5):358-62

152. Luo P, Liu Z, Zhao Y. Present situation of HIV, HCV and TP coinfection in HIV-infected patients in Liuzhou Area. Journal of Tropical Medicine. 2008;8(9):917-9

153. Yan YX, Gao YQ, Sun X, Wang W, Huang XJ, Zhang T, Li M, Zang CP, Li ZC, Wu H. Prevalence of hepatitis C virus and hepatitis B virus infections in HIV-positive Chinese patients. Epidemiology & Infection. 2011 Mar;139(3):354-60

154. Chu FY, Chiang SC, Su FH, Chang YY, Cheng SH. Prevalence of human immunodeficiency virus and its association with hepatitis B, C, and D virus infections among incarcerated male substance abusers in Taiwan. Journal of Medical Virology. 2009 Jun;81(6):973-8

155. Lee HC, Ko NY, Lee NY, Chang CM, Ko WC. Seroprevalence of viral hepatitis and sexually transmitted disease among adults with recently diagnosed HIV infection in Southern Taiwan, 2000-2005: upsurge in hepatitis C virus infections among injection drug users. Journal of the Formosan Medical Association. 2008 May;107(5):404-11

156. Sun HY, Lee HC, Liu CE, Yang CL, Su SC, Ko WC, Lin CY, Tsai JJ, Wong WW, Ho MW, Cheng SH, Lin YH, Miao WJ, Hung CC. Factors associated with isolated anti-hepatitis B core antibody in HIV-positive patients: impact of compromised immunity. Journal of Viral Hepatitis. 2010 Aug;17(8):578-87

157. Sun HY, Ko WC, Tsai JJ, Lee HC, Liu CE, Wong WW, Su SC, Ho MW, Cheng SH, Yang CH, Lin YH, Miao WJ, Sheng WH, Hung CC. Seroprevalence of chronic hepatitis B virus infection among taiwanese human immunodeficiency virus type 1-positive persons in the era of nationwide hepatitis B vaccination. American Journal of Gastroenterology. 2009 Apr;104(4):877-84

158. Jindal N, Arora U, Singh K. Prevalence of human immunodeficiency virus (HIV), hepatitis B virus, and hepatitis C virus in three groups of populations at high risk of HIV infection in Amritsar (Punjab), Northern India. Japanese Journal of Infectious Diseases. 2008 Jan;61(1):79-81

159. Kosaraju K, Padukone S, Bairy I. Co-infection with hepatitis viruses among HIV-infected individuals at a tertiary care centre in South India. Tropical Doctor. 2011 Jul;41(3):170-1

160. Sawant S, Agrawal S, Shastri J. Seroprevalence of Hepatitis B and Hepatitis C virus infection among HIV infected patients in Mumbai. Indian Journal of Sexually Transmitted Diseases. 2010 December;31(2):126

161. Saravanan S, Velu V, Kumarasamy N, Nandakumar S, Murugavel KG, Balakrishnan P, Suniti S, Thyagarajan SP. Coinfection of hepatitis B and hepatitis C virus in HIV-infected patients in south India. World Journal of Gastroenterology. 2007 Oct 7;13(37):5015-20

162. Alvarez-Uria G, Midde M, Pakam R, Naik PK. Gender differences, routes of transmission, sociodemographic characteristics and prevalence of HIV related infections of adults and children in an HIV cohort from a rural district of India. Infectious Disease Reports. 2012;4(1):66-70

163. Tankhiwale SS, Khadase RK, Jalgoankar SV. Seroprevalence of anti-HCV and hepatitis B surface antigen in HIV infected patients. Indian Journal of Medical Microbiology. 2003 October;21(4):268-70

164. Roche R, Amrita S, Nayak R. Prevalence of the Human Immunodeficiency Virus, the Hepatitis B Virus and the Hepatitis C Virus among the Patients at a Tertiary Health Care Centre: A Five Year Study. Journal of Clinical and Diagnostic Research. 2012;6(4 [Suppl-2]):623-6

165. Barman R, Islam S, Bowmick S, Dutta S. Is seroprevalence of hepatitis B and hepatitis C in Northeast India really high? Indian Journal of Gastroenterology. 2015;34(4):337-8

166. Anurag B, Pandey RP, Vivek R. A clinical study of prevalence of Hepatitis B virus, Hepatitis C virus and Syphilis (T. pallidum) in HIV positive patients. Scholars Journal of Applied Medical Sciences. 2015;3(3G):1490-6

167. Gupta S, Singh S. Hepatitis B and C virus co-infections in human immunodeficiency virus positive North Indian patients. World Journal of Gastroenterology. 2006 Nov 14;12(42):6879-83

168. Desai Praseeda S, Anuradha D, Shastri Jayanthi S. A study on the HBV and the HCV infections in female sex workers and their co-infection with HIV. Journal of Clinical and Diagnostic Research. 2013 01 Feb;7(2):234-7

169. Bhargava A, Singh DK, Rai R. Sero-prevalence of viral co-infections in HIV infected children of Northern India. Indian Journal of Pediatrics. 2009 Sep;76(9):917-9

170. Girish N, Nagarathnamma T, Venkatesha D. A study of hepatitis B virus co-infection in HIV infected patients. Indian Journal of Public Health Research and Development. 2015;6 (1):314-8

171. Sekar R, Amudhan M, Sivashankar M, Mythreyee M. Higher prevalence of sexually transmissible co-infections among the human immunodeficiency virus-infected population of South India. J Med Microbiol. 2011 Mar;60(Pt 3):394-5

172. Jayeeta S, Debraj S, Bhaswati B, Bibhuti S, Deepika K, Mazumder DNG, Runu C, Guha SK. Baseline characteristics of HIV & hepatitis B virus (HIV/HBV) co-infected patients from Kolkata, India. Indian Journal of Medical Research. 2016;143(5):636-42

173. Chalana H, Singh H, Sachdeva JK, Sharma S. Seroprevalence of human immunodeficiency virus, hepatitis B surface antigen, and hepatitis C in substance dependents admitted in a tertiary hospital at Amritsar, India. Asian J Psychiatr. 2013 Dec;6(6):552-5

174. Nelwan EJ, Van Crevel R, Alisjahbana B, Indrati AK, Dwiyana RF, Nuralam N, Pohan HT, Jaya I, Meheus A, Van Der Ven A. Human immunodeficiency virus, hepatitis B and hepatitis C in an Indonesian prison: prevalence, risk factors and implications of HIV screening. Tropical Medicine & International Health. 2010 Dec;15(12):1491-8

175. Lestari YD, Sitompul R, Edwar L, Djoerban Z. Ocular diseases among HIV/AIDS patients in Jakarta, Indonesia. Southeast Asian Journal of Tropical Medicine and Public Health. 2013;44(1):62-71

176. Anggorowati N, Yano Y, Heriyanto DS, Rinonce HT, Utsumi T, Mulya DP, Subronto YW, Hayashi Y. Clinical and virological characteristics of hepatitis B or C virus co-infection with HIV in Indonesian patients. Journal of Medical Virology. 2012 Jun;84(6):857-65

177. Fibriani A, Wisaksana R, Alisjahbana B, Indrati A, Schutten M, Crevel Rv, Ven Avd, Boucher CAB. Hepatitis B virus prevalence, risk factors and genotype distribution in HIV infected patients from West Java, Indonesia. Journal of Clinical Virology. 2014;59(4):235-41

178. Nissapatorn V, Kuppusamy I, Josephine FP, Jamaiah I, Rohela M, Khairul Anuar A. Tuberculosis: a resurgent disease in immunosuppressed patients. Southeast Asian Journal of Tropical Medicine & Public Health. 2006;37 Suppl 3:153-60

179. Akhtar A, Khan AH, Sulaiman SAS, Soo CT, Khan K. HBV and HIV co-infection: Prevalence and clinical outcomes in tertiary care hospital Malaysia. Journal of Medical Virology. 2016 01;88 (3):455-60

180. Zaw SK, Tun ST, Thida A, Aung TK, Maung W, Shwe M, Aye MM, Clevenbergh P. Prevalence of hepatitis C and B virus among patients infected with HIV: a cross-sectional analysis of a large HIV care programme in Myanmar. Tropical Doctor. 2013 Jul;43(3):113-5

181. Ionita G, Malviya A, Rajbhandari R, Schluter WW, Sharma G, Kakchapati S, Rijal S, Dixit S. Seroprevalence of hepatitis B virus and hepatitis C virus co-infection among people living with HIV/AIDS visiting antiretroviral therapy centres in Nepal: a first nationally representative study. International Journal of Infectious Diseases. 2017;60:64-9

182. Silverman JG, Decke MR, Gupta J, Dharmadhikari A, Seage GR, 3rd, Raj A. Syphilis and hepatitis B Co-infection among HIV-infected, sex-trafficked women and girls, Nepal. Emerging Infectious Diseases. 2008 Jun;14(6):932-4

183. Nafees M, Qasim A, Jafferi G, Anwar MS, Muazzam M. HIV infection, HIV/HCV and HIV/HBV co-infections among jail inmates of Lahore. Pakistan Journal of Medical Sciences. 2011 October;27(4):837-41

184. Sungkanuparph S, Wongprasit P, Manosuthi W, Atamasirikul K. Compliance with hepatitis B and hepatitis C virus infection screening among HIV-1 infected patients in a resource-limited setting. Southeast Asian Journal of Tropical Medicine & Public Health. 2008 Sep;39(5):863-6

185. Paris R, Sirisopana N, Benenson M, Amphaiphis R, Tuntichaivanich C, Myint KS, Brown AE. The association between hepatitis C virus and HIV-1 in preparatory cohorts for HIV vaccine trials in Thailand. AIDS. 2003 Jun 13;17(9):1363-7

186. Aurpibul L, Lumbiganon P, Kolasaraksa P, Hansudewechakul R, Sa-Nguanmoo P, Taeprasert P, Bunupuradah T, Poovorawan Y, Sirisanthana V, Puthanakit T. HIV and Hepatitis B coinfection among perinatally HIV-infected Thai adolescents. Pediatric Infectious Disease Journal. 2012 Sep;31(9):943-7

187. Law WP, Dore GJ, Duncombe CJ, Mahanontharit A, Boyd MA, Ruxrungtham K, Lange JM, Phanuphak P, Cooper DA. Risk of severe hepatotoxicity associated with antiretroviral therapy in the HIV-NAT Cohort, Thailand, 1996-2001. AIDS. 2003 Oct 17;17(15):2191-9

188. Jongjirawisan Y, Ungulkraiwit P, Sungkanuparph S. Isolated antibody to hepatitis B core antigen in HIV-1 infected patients and a pilot study of vaccination to determine the anamnestic response. Journal of the Medical Association of Thailand. 2006 Dec;89(12):2028-34

189. Sirinak C, Kittikraisak W, Pinjeesekikul D, Charusuntonsri P, Luanloed P, Srisuwanvilai LO, Nateniyom S, Akksilp S, Likanonsakul S, Sattayawuthipong W, Burapat C, Varma JK. Viral hepatitis and HIV-associated tuberculosis: risk factors and TB treatment outcomes in Thailand. BMC Public Health. 2008;8:245

190. Sungkanuparph S, Vibhagool A, Manosuthi W, Kiertiburanakul S, Atamasirikul K, Aumkhyan A, Thakkinstian A. Prevalence of hepatitis B virus and hepatitis C virus co-infection with human immunodeficiency virus in Thai patients: a tertiary-care-based study. Journal of the Medical Association of Thailand. 2004 Nov;87(11):1349-54

191. Chotiprasitsakul D, Wongprasit P, Atamasirikul K, Sungkanuparph S. Screening of hepatitis B virus infection among HIV-infected patients receiving antiretroviral therapy. Journal of Infectious Diseases and Antimicrobial Agents. 2010;27(2):69-75

192. Akekawatchai C, Sretapunya W, Pipatsatitpong D, Chuenchit T. Hepatitis B or C virus coinfection in and risks for transaminitis in human immunodeficiency virus - infected thais on combined antiretroviral therapy. Asian Biomedicine. 2015;9(3):353-61

193. Manosuthi W, Sukasem C, Lueangniyomkul A, Mankatitham W, Thongyen S, Nilkamhang S, Manosuthi S, Sungkanuparph S. CYP2B6 haplotype and biological factors responsible for hepatotoxicity in HIV-infected patients receiving efavirenz-based antiretroviral therapy. International Journal of Antimicrobial Agents. 2014 Mar;43(3):292-6

194. Nguyen CH, Ishizaki A, Chung PT, Hoang HT, Nguyen TV, Tanimoto T, Lihana R, Matsushita K, Bi X, Pham TV, Ichimura H. Prevalence of HBV infection among different HIV-risk groups in Hai Phong, Vietnam. Journal of Medical Virology. 2011 Mar;83(3):399-404

195. Rangarajan S, Colby DJ, Truong GL, Huu HN, Thu VTT, Quoc BL, Broh TP, Tri DT, Giang DD, Chen M, Zeng YW, West G. Factors associated with HIV RNA viral loads in ART-naive patients: implications for treatment as prevention in concentrated epidemics. Journal of Virus Eradication. 2016 Jan;2(1):36-42

196. Lee SH, Kim KH, Lee SG, Choy H, Chen DH, Chung JS, Kwak IS, Cho GJ. Causes of death and risk factors for mortality among hivinfected patients receiving antiretroviral therapy in korea. Journal of Korean Medical Science. 2013 July;28(7):990-7

197. de los Angeles Pando M, Biglione MM, Toscano MF, Rey JA, Russell KL, Negrete M, Gianni S, Martinez-Peralta L, Salomon H, Sosa-Estani S, Montano SM, Olson JG, Sanchez JL, Carr JK, Avila MM. Human immunodeficiency virus type 1 and other viral co-infections among young heterosexual men and women in Argentina. Am J Trop Med Hyg. 2004 Aug;71(2):153-9

198. Laufer N, Quarleri J, Bouzas MB, Juncos G, Cabrini M, Moretti F, Bolcic F, Fernandez-Giuliano S, Mammana L, Salomon H, Cahn P. Hepatitis B virus, hepatitis C virus and HIV coinfection among people living with HIV/AIDS in Buenos Aires, Argentina. Sexually Transmitted Diseases. 2010 May;37(5):342-3

199. Bissio E, Lopardo GD. Incidence of hyperbilirubinemia and jaundice due to atazanavir in a cohort of Hispanic patients. AIDS Research and Human Retroviruses. 2013;29(3):415-7

200. Carobene M, Bolcic F, Farias MS, Quarleri J, Avila MM. HIV, HBV, and HCV molecular epidemiology among trans (transvestites, transsexuals, and transgender) sex workers in Argentina. Journal of Medical Virology. 2014 Jan;86(1):64-70

201. Neto WK, Sanabani SS, Jamal LF, Sabino EC. Prevalence, risk factors and genetic characterization of human T-cell lymphotropic virus types 1 and 2 in patients infected with human immunodeficiency virus type 1 in the Cities of Ribeirao Preto and Sao Paulo. [Portuguese]

Prevalencia, fatores de risco e caracterizacao genetica dos virus linfotropico de celulas T humana tipo 1 e 2 em pacientes infectados pelo virus da imunodeficiencia humana tipo 1 nas Cidades de Ribeirao Preto e Sao Paulo. Revista da Sociedade Brasileira de Medicina Tropical. 2009;42(3):264-70

202. Pavan MH, Aoki FH, Monteiro DT, Gonçales NS, Escanhoela CA, Júnior FL. Viral hepatitis in patients infected with human immunodeficiency virus. Brazilian Journal of Infectious Diseases. 2003 2003;7(4):253-61

203. de Almeida Pereira RA, Mussi AD, de Azevedo e Silva VC, Souto FJ. Hepatitis B Virus infection in HIV-positive population in Brazil: results of a survey in the state of Mato Grosso and a comparative analysis with other regions of Brazil. BMC Infectious Diseases. 2006;6:34

204. Vieira AC, Tizzot MRPA, Santos VLP, Bovo F, Reason IM. Epidemiological analysis of serological markers of hepatitis B in HIV+ patients from Curitiba and metropolitan region. Jornal Brasileiro de Patologia e Medicina Laboratorial. 2015;51(1):17-21

205. Zago AM, Machado TF, Cazarim FL, Miranda AE. Prevalence and risk factors for chronic hepatitis B in HIV patients attended at a sexually-transmitted disease clinic in Vitoria, Brazil^ien. Braz j infect dis. 2007 10;11(5):475-8

206. Grinsztejn B, Bastos FI, Veloso VG, Friedman RK, Pilotto JH, Schechter M, Derrico M, Andrade A, Lourenco MC, Moreira RI, Russomano F, Morgado M, Currier JS. Assessing sexually transmitted infections in a cohort of women living with HIV/AIDS, in Rio de Janeiro, Brazil. International Journal of STD & AIDS. 2006 Jul;17(7):473-8

207. Kreitchmann R, Fuchs SC, Suffert T, Preussler G. Perinatal HIV-1 transmission among low income women participants in the HIV/AIDS Control Program in Southern Brazil: a cohort study. Bjog. 2004 Jun;111(6):579-84

208. Reiche EM, Bonametti AM, Morimoto HK, Morimoto AA, Wiechemann SL, Matsuo T, Vissoci Reiche F, Vogler IH. Epidemiological, immunological and virological characteristics, and disease progression of HIV-1/HCV-co-infected patients from a southern Brazilian population. Int J Mol Med. 2008 Mar;21(3):387-95

209. Aires RS, Matos MAD, Lopes CLR, Teles SA, Kozlowski AG, Silva AMC, Filho JAA, Lago BV, Mello FCA, Martins RMB. Prevalence of hepatitis B virus infection among tuberculosis patients with or without HIV in Goiânia City, Brazil. Journal of clinical virology: the official publication of the Pan American Society for Clinical Virology. 2012 01/01/

210. Braga WS, da Costa Castilho M, dos Santos IC, Moura MA, Segurado AC. Low prevalence of hepatitis B virus, hepatitis D virus and hepatitis C virus among patients with human immunodeficiency virus or acquired immunodeficiency syndrome in the Brazilian Amazon basin. Revista Da Sociedade Brasileira de Medicina Tropical. 2006 Nov-Dec;39(6):519-22

211. Morimoto HK, Caterino-De-Araujo A, Morimoto AA, Reiche EM, Ueda LT, Matsuo T, Stegmann JW, Reiche FV. Seroprevalence and risk factors for human T cell lymphotropic virus type 1 and 2 infection in human immunodeficiency virus-infected patients attending AIDS referral center health units in Londrina and other communities in Parana, Brazil. AIDS Res Hum Retroviruses. 2005 Apr;21(4):256-62

212. Carmo RA, Guimaraes MD, Moura AS, Neiva AM, Versiani JB, Lima LV, Freitas LP, Rocha MO. The influence of HCV coinfection on clinical, immunological and virological responses to HAART in HIV-patients. Brazilian Journal of Infectious Diseases. 2008 Jun;12(3):173-9

213. Galisteu KJ, Cardoso LV, Furini AAD, Schiesari A, Cesarino CB, Franco C, Baptista ARD, Machado RLD. Opportunistic infections among individuals with HIV-1/AIDS in the highly active antiretroviral therapy era at a Quaternary Level Care Teaching Hospital. Revista Da Sociedade Brasileira De Medicina Tropical. 2015 Mar-Apr;48(2):149-56

214. Barbosa JR, Bezerra CS, Carvalho-Costa FA, Azevedo CPd, Flores GL, Colares JKB, Lima DM, Lampe E, Villar LM. Cross-sectional study to determine the prevalence of hepatitis B and C virus infection in high risk groups in the northeast region of Brazil. International Journal of Environmental Research and Public Health. 2017;14(7):793

215. Portelinha Filho AM, Nascimento CUd, Tannouri TN, Troiani C, Ascêncio EL, Bonfim R, D'Andrea LAZ, Prestes-Carneiro LE. Seroprevalence of HBV, HCV and HIV co-infection in selected individuals from state of São Paulo, Brazil^ien. Mem Inst Oswaldo Cruz. 2009 11;104(7):960-3

216. De Souza NC, Botelho CA, Honer MR. Retrospective study of a pioneer antenatal screening program with 8,477 pregnant women in Brazil. Clin Exp Obstet Gynecol. 2004;31(3):217-20

217. Oliveira MP, Lemes PS, Matos MAD, Del-Rios NHA, Santos Carneiro MA, Costa Silva AM, Lopes CLR, Teles SA, Aires RS, Lago BV, Araujo NM, Martins RMB. Overt and occult hepatitis B virus infection among treatment-naive HIV-infected patients in Brazil. Journal of Medical Virology. 2016 01;88 (7):1222-9

218. Reiche EM, Bonametti AM, Watanabe MA, Morimoto HK, Morimoto AA, Wiechmann SL, Bregano JW, Matsuo T, Reiche FV. Socio-demographic and epidemiological characteristics associated with human immunodeficiency virus type I (HIV-1) infection in HIV-1-exposed but uninfected individuals, and in HIV-1-infected patients from a southern Brazilian population. Rev Inst Med Trop Sao Paulo. 2005 Sep-Oct;47(5):239-46

219. Brandao NAA, Pfrimer IAH, Martelli CMT, Turchi MD. Prevalence of hepatitis B and C infection and associated factors in people living with HIV in midwestern Brazil. Brazilian Journal of Infectious Diseases. 2015;19(4):426-30

220. Soares Sampaio A, Arraes de Alencar LC, Moura PMMFd, Barros Correia Jd, Barros Barreto Sd, Castelo A. Prevalencia de la co-infección con hepatitis B y C en pacientes HIV positivos y factores de riesgo asociados^ies. Actual SIDA. 2009 03;17(63):12-7

221. Martins S, do Livramento A, Andrigueti M, Kretzer IF, Machado MJ, Spada C, Treitinger A. The prevalence of hepatitis B virus infection markers and socio-demographic risk factors in HIV-infected patients in Southern Brazil. Revista da Sociedade Brasileira de Medicina Tropical. 2014 01 Sep;47(5):552-8

222. Freitas SZ, Soares CC, Tanaka TSO, Lindenberg ASC, Teles SA, Torres MS, Mello FCA, Mendes-Correa MC, Savassi-Ribas F, Motta-Castro ARC. Prevalence, risk factors and genotypes of hepatitis B infection among HIV-infected patients in the State of MS, Central Brazil. Brazilian Journal of Infectious Diseases. 2014 01 Sep;18(5):473-80

223. Tavora LG, Hyppolito EB, Cruz JN, Portela NM, Pereira SM, Veras CM. Hepatitis B, C and HIV co-infections seroprevalence in a northeast Brazilian center. Arq Gastroenterol. 2013 Oct-Dec;50(4):277-80

224. Hoyos-Orrego A, Massaro-Ceballos M, Ospina-Ospina M, Gomez-Builes C, Vanegas-Arroyave N, Tobon-Pereira J, Jaramillo-Hurtado J, Rugeles-Lopez MT. Serological markers and risk factors for hepatitis B and C viruses in patients infected with human immunodeficiency virus. Rev Inst Med Trop Sao Paulo. 2006 Nov-Dec;48(6):321-6

225. Andrade-Villanueva J, Gonzalez-Hernandez L, Llamas-Covarrubias M, Ponce-Herrera D, Soria-Rodriguez R, Zuniga-Quinonez S, Martinez-Ayala P. Evaluation of HIV-infection control, risk behaviours and comorbidities among inmates in Mexico. Journal of the International AIDS Society. 2015 April;Conference: HIV Drug Therapy in the Americas 2015. Mexico City Mexico. Conference Publication: (var.pagings). 18 (SUPPL. 2):14

226. Jose-Abrego A, Panduro A, Fierro NA, Roman S. High prevalence of HBV infection, detection of subgenotypes F1b, A2, and D4, and differential risk factors among Mexican risk populations with low socioeconomic status. Journal of Medical Virology. 2017;89(12):2149-57

227. Luis J, Felipe U, Patricia I, Veronica R, Yazmin M, Andrea G. Epidemiological characteristics of attendants of VCT in the context of a faster and integral diagnosis of HIV/STI in Condesa Clinic, Mexico City. Journal of the International AIDS Society. 2014 May;17:7

228. Jayaraman GC, Bush KR, Lee B, Singh AE, Preiksaitis JK. Magnitude and determinants of first-time and repeat testing among individuals with newly diagnosed HIV infection between 2000 and 2001 in Alberta, Canada: results from population-based laboratory surveillance. Journal of Acquired Immune Deficiency Syndromes: JAIDS. 2004 Dec 15;37(5):1651-6

229. Pittman C, Plitt S, Birse T, Doucette K, Romanowski B, Cooper R, Houston S, Shafran S, Singh AE. Prevalence and correlates of HIV and hepatitis B virus coinfection in Northern Alberta. Canadian Journal of Infectious Diseases and Medical Microbiology. 2014 January-February;25(1):e8-e13

230. Ohl M, Tate J, Duggal M, Skanderson M, Scotch M, Kaboli P, Vaughan-Sarrazin M, Justice A. Rural residence is associated with delayed care entry and increased mortality among veterans with human immunodeficiency virus infection. Med Care. 2010 Dec;48(12):1064-70

231. Gowda C, Brown TT, Compher C, Forde KA, Kostman J, Shaw PA, Tien PC, Io Re V, III. Prevalence and predictors of low muscle mass in HIV/viral hepatitis coinfection. AIDS. 2016;30(16):2519-28

232. Yacisin K, Maida I, Rios MJ, Soriano V, Nunez M. Hepatitis C virus coinfection does not affect CD4 restoration in HIV-infected patients after initiation of antiretroviral therapy. AIDS Res Hum Retroviruses. 2008 Jul;24(7):935-40

233. Prussing C, Chan C, Pinchoff J, Kersanske L, Bornschlegel K, Balter S, Drobnik A, Fuld J. HIV and viral hepatitis co-infection in New York City, 2000-2010: prevalence and case characteristics. Epidemiology & Infection. 2015;143(7):1408-16

234. Toussi SS, Abadi J, Rosenberg M, Levanon D. Prevalence of hepatitis B and C virus infections in children infected with HIV. Clinical Infectious Diseases. 2007 Sep 15;45(6):795-8

235. Tien PC, Kotler DP, Overton ET, Lewis CE, Rimland D, Bacchetti P, Scherzer R, Gripshover B, Study Fat Redistribution M. Regional adipose tissue and elevations in serum aminotransferases in HIV-infected individual. Jaids-Journal of Acquired Immune Deficiency Syndromes. 2008 Jun;48(2):169-76

236. Osborn MK, Guest JL, Rimland D. Hepatitis B virus and HIV coinfection: relationship of different serological patterns to survival and liver disease. HIV Medicine. 2007 Jul;8(5):271-9

237. Hendricks KM, Erzen HD, Wanke CA, Tang AM. Nutrition issues in the HIV-infected injection drug user: findings from the nutrition for healthy living cohort. J Am Coll Nutr. 2010 Apr;29(2):136-43

238. Reingold JS, Wanke C, Kotler DP, Lewis CE, Tracy R, Heymsfield S, Tien PC, Bacchetti P, Scherzer R, Grunfeld C, Shlipak MG. Association of HIV infection and HIV/HCV coinfection with C-reactive protein levels - The Fat Redistribution and Metabolic Change in HIV Infection (FRAM) study. Jaids-Journal of Acquired Immune Deficiency Syndromes. 2008 Jun;48(2):142-8

239. Psevdos G, Jr., Kim JH, Suh JS, Sharp VL. Predictors of loss of hepatitis B surface antigen in HIV-infected patients. World Journal of Gastroenterology. 2010 Mar 7;16(9):1093-6

240. Tien PC, Bacchetti P, Gripshover B, Overton ET, Rimland D, Kotler D, Fat R, Metabolic Change in HIVISI. Association between hepatitis C virus coinfection and regional adipose tissue volume in HIV-infected men and women. Journal of Acquired Immune Deficiency Syndromes: JAIDS. 2007 May 1;45(1):60-5

241. Santiago-Munoz P, Roberts S, Sheffield J, McElwee B, Wendel GD, Jr. Prevalence of hepatitis B and C in pregnant women who are infected with human immunodeficiency virus. American Journal of Obstetrics & Gynecology. 2005 Sep;193(3 Pt 2):1270-3

242. Ofotokun I, Smithson SE, Lu C, Easley KA, Lennox JL. Liver enzymes elevation and immune reconstitution among treatment-naive HIV-infected patients instituting antiretroviral therapy. Am J Med Sci. 2007 Nov;334(5):334-41

243. Glesby MJ, Hoover DR, Raiszadeh F, Lee I, Shi Q, Milne G, Sanchez SC, Gao W, Kaplan RC, Morrow JD, Anastos K. Oxidant stress in HIV-infected women from the Women's Interagency HIV Study. Antiviral Therapy. 2009;14(6):763-9

244. Glesby MJ, Hoover DR, Shi Q, Danoff A, Howard A, Tien P, Merenstein D, Cohen M, Golub E, Dehovitz J, Nowicki M, Anastos K. Glycated haemoglobin in diabetic women with and without HIV infection: data from the Women's Interagency HIV Study. Antiviral Therapy. 2010;15(4):571-7

245. Al-Harthi L, Voris J, Du W, Wright D, Nowicki M, Frederick T, Landay A, Kovacs A. Evaluating the impact of hepatitis C virus (HCV) on highly active antiretroviral therapy-mediated immune responses in HCV/HIV-coinfected women: role of HCV on expression of primed/memory T cells. Journal of Infectious Diseases. 2006 May 1;193(9):1202-10

246. Mehta SH, Moore RD, Thomas DL, Chaisson RE, Sulkowski MS. The effect of HAART and HCV infection on the development of hyperglycemia among HIV-infected persons. Journal of Acquired Immune Deficiency Syndromes: JAIDS. 2003 Aug 15;33(5):577-84

247. Kim JH, Psevdos G, Suh J, Sharp VL. Co-infection of hepatitis B and hepatitis C virus in human immunodeficiency virus-infected patients in New York City, United States. World Journal of Gastroenterology. 2008 Nov 21;14(43):6689-93

248. Weiss ES, Cornwell EE, 3rd, Wang T, Syin D, Millman EA, Pronovost PJ, Chang D, Makary MA. Human immunodeficiency virus and hepatitis testing and prevalence among surgical patients in an urban university hospital. Am J Surg. 2007 Jan;193(1):55-60

249. Castaneda-Sceppa C, Bermudez OI, Wanke C, Forrester JE. Predictors of insulin resistance among Hispanic adults infected with or at risk of infection with the human immunodeficiency virus and hepatitis C virus. Journal of Viral Hepatitis. 2008 Dec;15(12):878-87

250. Visnegarwala F, Chen L, Raghavan S, Tedaldi E. Prevalence of diabetes mellitus and dyslipidemia among antiretroviral nave patients co-infected with hepatitis C virus (HCV) and HIV-1 compared to patients without co-infection. Journal of Infection. 2005 May;50(4):331-7

251. Anderson KB, Guest JL, Rimland D. Hepatitis C virus coinfection increases mortality in HIV-infected patients in the highly active antiretroviral therapy era: data from the HIV Atlanta VA Cohort Study. Clinical Infectious Diseases. 2004 Nov 15;39(10):1507-13

252. Sullivan PS, Hanson DL, Teshale EH, Wotring LL, Brooks JT. Effect of hepatitis C infection on progression of HIV disease and early response to initial antiretroviral therapy. AIDS. 2006 May 12;20(8):1171-9

253. Crowell TA, Berry SA, Fleishman JA, LaRue RW, Korthuis PT, Nijhawan AE, Moore RD, Gebo KA. Impact of hepatitis coinfection on healthcare utilization among persons living with HIV. JAIDS, Journal of Acquired Immune Deficiency Syndromes. 2015;68(4):425-31

254. Weiss JJ, Osorio G, Ryan E, Marcus SM, Fishbein DA. Prevalence and patient awareness of medical comorbidities in an urban AIDS clinic. AIDS Patient Care STDS. 2010 Jan;24(1):39-48

255. Ireland J, Cheng DM, Samet JH, Bridden C, Quinn E, Saitz R. Operating characteristics of carbohydrate-deficient transferrin (CDT) for identifying unhealthy alcohol use in adults with HIV infection. AIDS Care. 2011 Nov;23(11):1483-91

256. Cohen MH, French AL, Benning L, Kovacs A, Anastos K, Young M, Minkoff H, Hessol NA. Causes of death among women with human immunodeficiency virus infection in the era of combination antiretroviral therapy. Am J Med. 2002 Aug 1;113(2):91-8
