# Supplementary material for: Prevalence and burden of HBV co‐infection among people living with HIV: A global systematic review and meta‐analysis
Source: J Viral Hepat. 2019 Dec 22;27(3):294–315. doi: 10.1111/jvh.13217 (PMC7383613; doi:10.1111/jvh.13217)
Supplement: Supplementary file 3 [file JVH-27-294-s003.docx]

**Supplementary Table S4:** Meta-analyses comparing HBsAg prevalence among HIV positive and negative samples among selected populations by country and region

| **Region** | **Country** | **All studies** | | **Best estimates prevalence (Range) and characteristics of study design and Sample** | | | | | | |
| --- | --- | --- | --- | --- | --- | --- | --- | --- | --- | --- |
|  |  | **N** | **Total** | **HIV** | **HBsAG** | **HBsAg/HIV** | **Odds Ratio (95% CI)** | **PWID** | **Score** | **Year** |
| **General population** |  |  |  |  |  |  |  |  |  |  |
| West and Central Africa | Burkina Faso^1^ | 1 | 336 | 5.4 | 9.5 | 0.5 | 0.0 (0.0-0.1) |  | C3 | 2005 |
|  | Cameroon^2,3^ | 2 | 22724 | 3.3 (3.3-31.5) | 12.0 (6.3-12.0) | 17.3 (4.2-17.3) | 1.3 (1.2-1.4) |  | B3 | 2013 |
|  | Eqt. Guinea^4^ | 1 | 2937 | 7.8 | 8.8 | 15.7 | 1.8 (1.2-2.6) |  | B3 | 2013 |
|  | Ghana^5-8^ | 4 | 20234 | 4.0 (4.0-7.7) | 12.4 (7.1-12.4) | 6.0 (2.4-18.7) | 0.5 (0.4-0.7) |  | C2 | 2007 |
|  | Mali^9^ | 1 | 11592 | 4.5 | 13.7 | 25.3 | 2.0 (1.6-2.5) |  | C2 | 2002 |
|  | Nigeria^10-17^ | 8 | 26741 | 12.9 (3.0-52.1) | 6.8 (3.4-19.4) | 1.3 (0.0-19.8) | 1.3 (1.1-1.5) |  | B2 | 2016 |
| South Africa | Zimbabwe^18^ | 1 | 369 | 20.1 | 3 | 1.4 | 0.4 (0.0-2.8) |  | C2 | 2005 |
| East Africa | Djibouti^19^ | 1 | 9210 | 1.9 | 10 | 9.7 | 1.0 (0.6-1.6) |  | C3 | 2000 |
|  | Ethiopia^20-23^ | 4 | 19351 | 1.6 (1.6-11.9) | 4.7 (2.0-8.3) | 7.9 (0.7-61.4) | 3.1 (2.3-4.3) |  | B2 | 2013 |
|  | Kenya^24^ | 1 | 418 | 20.4 | 28 | 20.9 | 0.1 (0.0-0.1) |  | B1 | 2007 |
|  | Mozambique^25,26^ | 2 | 2257 | 8.5 (8.5-13.8) | 9.4 (6.8-9.4) | 13.8 (10.1-13.8) | 2.1 (1.5-3.0) | 0 | C0 | 2009 |
|  | Rwanda^27^ | 1 | 1233 | 31.1 | 2.4 | 5.7 | 1.7 (0.9-2.9) |  | C2 | 2001 |
|  | Uganda^28-31^ | 4 | 11049 | 1.9 (1.9-50.0) | 3.4 (1.4-9.8) | 6.0 (4.6-8.3) | 0.8 (0.6-1.1) |  | B2 | 1999 |
| South America | Brazil^32-34^ | 3 | 1469278 | 0.3 (0.1-0.3) | 0.4 (0.1-1.1) | 0.5 (0.5-1.0) | 7.8 (4.7-13.1) |  | B2 | 2012 |
|  | Colombia^35^ | 1 | 54499 | 0.5 | 0.2 | 1.2 | 5.2 (1.6-16.3) |  | C3 | 2009 |
| South Asia and SEA | India^36-40^ | 5 | 196091 | 0.3 (0.1-1.0) | 1.6 (1.0-3.5) | 8.3 (0.0-8.3) | 2.2 (1.4-3.4) |  | B2 | 2017 |
| North Africa/Middle East | Egypt^41^ | 1 | 66234 | 0.1 | 0.5 | 3.4 | 7.7 (1.9-31.5) |  | C2 | 2015 |
| **Total (median, IQR)** |  |  |  | **4.5 (1.6-10.4)** | **6.1 (2.0-9.5)** | **6.0 (1.4-10.1)** | **1.3 (0.91-1.85)** |  |  |  |
| **Heterosexual/PLHIV** |  |  |  |  |  |  |  |  |  |  |
| West and Central Africa | Cameroon^42^ | 1 | 959 | 8.4 | 7 | 8.8 | 1.2 (0.5-2.6) |  | B2 | 2012 |
| **Total (median, IQR)** |  |  |  | **8.4** | **7.0** | **8.0** | **1.2 (0.5-2.6)** |  |  |  |
| **Children** |  |  |  |  |  |  |  |  |  |  |
| South Africa | South Africa^43,44^ | 2 | 1303 | 61.4 (6.1-61.4) | 0.0 (0.0-0.3) | 0.5 (0.0-0.5) | 2.1 (0.3-15.3) |  | B3 | 2014 |
| East Africa | Tanzania^45^ | 1 | 547 | 28.7 | 0.9 | 7 | 5.8 (2.0-17.0) |  | B2 | 2009 |
| **Total (median, IQR)** |  |  |  | **28.7 (6.1-61.4)** | **0.3 (0.0-0.9)** | **0.5 (0.0-7.0)** | **4.6 1.8-11.8)** |  |  |  |
| **High risk** |  |  |  |  |  |  |  |  |  |  |
| West and Central Africa | Nigeria^16,46^ | 2 | 634 | 34.2 (11.7-34.2) | 10.4 (4.2-10.4) | 21.2 (21.2-35.7) | 5.5 (3.2-9.4) |  | B2 | 2013 |
| East Africa | Ethiopia^47^ | 1 | 620 | 49.2 | 2.7 | 4 | 0.7 (0.3-1.5) | 14.2 | B0 | 2007 |
|  | Kenya^48^ | 1 | 752 | 39.4 | 1.3 | 6.8 | 3.2 (1.5-7.0) | 49.0 | B1 | 2014 |
|  | Malawi^49^ | 1 | 164 | 36.6 | 2.4 | 0.6 | 0.4 (0.0-3.9) |  | B2 | 2006 |
|  | Mauritius^50^ | 1 | 299 | 32.7 | 0 | 0 | 0.0 (0.0-0.0) | 40.1 | B2 | 2010 |
| Latin America | Argentina^51^ | 1 | 273 | 34.1 (34.1-34.1) | 37.7 | 3.2 | 0.0 (0.0-0.1) |  | B2 | 2009 |
|  | Brazil^52,53^ | 2 | 1630 | 27.6 (24.7-27.6) | 2.2 (2.2-5.5) | 3.6 (1.6-3.6) | 0.4 (0.2-0.8) |  | B2 | 2010 |
| North America | Mexico^54,55^ | 2 | 11169 | 35.6 (20.0-35.6) | 6.7 (0.3-6.7) | 29.4 (3.4-29.4) | 7.1 (5.4-9.4) | 12.5 | B2 | 2015 |
|  | USA^56^ | 1 | 2734 | 35 | 23.4 | 31 | 0.8 (0.7-0.9) | 24.0 | B2 | 2004 |
| South Asia and SEA | India^57-59^ | 3 | 25433 | 42.4 (1.3-42.4) | 4.8 (1.1-5.9) | 7.0 (1.6-18.3) | 3.7 (2.4-5.8) |  | B3 | 2007 |
|  | Indonesia^60^ | 1 | 696 | 9.1 | 5.5 | 3.4 | 0.5 (0.1-2.2) |  | B2 | 2009 |
|  | Thailand^61^ | 1 | 381 | 42.2 | 2.6 | 9.9 | 2.0 (0.9-4.4) | 7.3 | C2 | 2001 |
|  | Vietnam^62^ | 1 | 1355 | 22.2 | 9.7 | 10.3 | 0.8 (0.5-1.2) | 56.1 | B2 | 2007 |
| W. Europe | Ireland^63^ | 1 | 8839 | 1.1 | 0.5 | 2.1 | 4.4 (1.0-18.3) | 15.5 | B0 | 2015 |
|  | Spain^64^ | 1 | 1496 | 10.4 | 7.5 | 9 | 1.1 (0.6-2.0) |  | B2 | 2008 |
| East Asia | China^65^ | 1 | 620 | 49.2 | 3.5 | 7.5 | 1.1 (0.6-2.0) |  | C3 | 2005 |
|  | Taiwan^66,67^ | 2 | 1128 | 53.2 (52.8-53.2) | 5.3 (5.3-5.3) | 18.9 (18.9-18.9) | 1.8 (1.3-2.6) | 100 | A2 | 2010 |
| **Total (median, IQR)** |  |  |  | **30.1 (10.4-36.6)** | **4.5 (2.2-7.5)** | **6.9(3.2-11.5)** | **1.15 (0.64-2.1)** |  |  |  |
| **PWID** |  |  |  |  |  |  |  |  |  |  |
| South Asia and SEA | India^68^ | 1 | 2292 | 25.3 | 6.3 | 12.9 | 1.6 (1.2-2.1) | 100 | B2 | 2014 |
|  | Myanmar^69^ | 1 | 318 | 27 | 31.8 | 41.9 | 0.9 (0.6-1.5) | 100 | B2 | 2009 |
|  | Vietnam^70,71^ | 2 | 3010 | 28.2 (28.2-29.4) | 9.5 (9.5-9.5) | 16.3 (9.4-16.3) | 0.8 (0.6-0.9) |  | A3 | 2009 |
| North Africa/Middle East | Iran (Islamic Republic of)^72,73^ | 2 | 622 | 15.2 (15.2-67.5) | 3.1 (0.0-3.1) | 4.3 (4.3-44.2) | 10.6 (5.8-19.3) | 100 | B2 | 2002 |
|  | Libya^74^ | 1 | 328 | 89.6 | 0.3 | 5.1 | 1.8 (0.2-13.9) | 100 | B0 | 2010 |
| East Asia | China^69,75,76^ | 3 | 2889 | 47.3 (25.5-47.3) | 29.3 (18.9-31.5) | 15.1 (1.1-59.6) | 0.3 (0.3-0.4) | 100 | B3 | 2003 |
|  | Taiwan^77^ | 1 | 753 | 7 | 16.1 | 11.5 | 0.6 (0.3-1.5) | 100 | B2 | 2005 |
| **Total (median, IQR)** |  |  |  | **31.6 (26.3-53.0)** | **6.3 (3.1-29.3)** | **15.7 (7.3-30.4)** | **1.4 (0.7-2.8)** |  |  |  |
| **MSM** |  |  |  |  |  |  |  |  |  |  |
| East Africa | Tanzania^78^ | 1 | 509 | 12.8 | 3.5 | 9.2 | 2.4 (0.9-6.3) | 13.0 | B0 | 2007 |
| South America | Brazil^79^ | 1 | 1165 | 75.9 | 13.6 | 31 | 0.3 (0.3-0.5) |  | B0 | 1998 |
| South America | Peru^80^ | 1 | 2703 | 12.5 | 2 | 9.5 | 4.4 (2.8-6.9) |  | B0 | 2003 |
| North America | Canada^81^ | 1 | 442 | 66.5 | 0.2 | 2.8 | 4.1 (0.5-33.2) |  | B2 | 2012 |
|  | USA^82^ | 1 | 1819 | 31.6 | 0.9 | 4.7 | 3.6 (1.9-6.6) | 1.0 | B2 | 2003 |
| Asia Pacific & Australasia | Japan^83^ | 1 | 7898 | 1.7 | 1.5 | 17.9 | 8.2 (4.7-14.5) | 0 | B2 | 2011 |
| **Total (median, IQR)** |  |  |  | **22.2 (12.5-66.5)** | **1.8 (0.9-3.5)** | **9.3 (4.7-17.9)** | **2.7 (0.7-10.2)** |  |  |  |
| **Global** |  | **85** | **311719** |  |  |  | **1.42 (1.1-1.8)** |  |  |  |

**References**

1. Simpore J, Ilboudo D, Karou D, Pietra V, Granato M, Esposito M, Santarelli R, Pignatelli S, Bere A, Angeloni A. Prevalence of HHV-8 infections associated with HIV, HBV and HCV in pregnant women in Burkina Faso. Journal of Medical Sciences. 2006;6(1):93-8

2. Rodgers MA, Vallari AS, Harris B, Yamaguchi J, Holzmayer V, Forberg K, Berg MG, Kenmenge J, Ngansop C, Awazi B, Mbanya D, Kaptue L, Brennan C, Cloherty G, Ndembi N. Identification of rare HIV-1 Group N, HBV AE, and HTLV-3 strains in rural South Cameroon. Virology. 2017;504:141-51

3. Ankouane F, Noah DN, Atangana MM, Simo RK, Guekam PR, Sida MB. Seroprevalence of hepatitis B and C viruses, HIV-1/2 and syphilis among blood donors in the Yaounde Central Hospital in the centre region of Cameroon. Transfusion Clinique Et Biologique. 2016 May;23(2):72-7

4. Xie D, Li J, Chen J, Eyi UM, Matesa RA, Obono MMO, Ehapo CS, Yang L, Yang H, Yang H, Lin M. Seroprevalence of human immunodeficiency virus, hepatitis B virus, hepatitis C virus, and Treponema pallidum infections among blood donors on Bioko Island, Equatorial Guinea. PLoS ONE. 2015;10(10):e0139947

5. Lokpo SY, Dakorah MP, Norgbe GK, Osei-Yeboah J, Adzakpah G, Sarsah I, Deku JG, Afeke I, Asiamah EA, Manaphraim NYB, Asare I, Ayidzoe BJ, Allotey EA, Nani EA, Amoah P. The Burden and Trend of Blood-Borne Pathogens among Asymptomatic Adult Population in Akwatia: A Retrospective Study at the St. Dominic Hospital, Ghana. Journal of Tropical Medicine. 2017

6. Walana W, Ahiaba S, Hokey P, Vicar EK, Acquah SEK, Der EM, Ziem JB. Sero-prevalence of HIV, HBV and HCV among blood donors in the Kintampo municipal hospital, Ghana. British Microbiology Research Journal. 2014;4(12):1491-9

7. Cho Y, Bonsu G, Akoto-Ampaw A, Nkrumah-Mills G, Nimo JJA, Park JK, Ki M. The prevalence and risk factors for hepatitis B surface Ag positivity in pregnant women in eastern region of Ghana. Gut and Liver. 2012 April;6(2):235-40

8. Amidu N, Owiredu W, Addai-Mensah O, Alhassan A, Quaye L, Batong B. Seroprevalence and Risk Factors for Human Immunodeficiency Virus, Hepatitis B and C Vi-ruses Infections among Blood Donors at the Bolgatanga Regional Hospital in Bolgatanga, Ghana. Ghana Science Association Journal. 2010 07/20/;12(1)

9. Tounkara A, Sarro YS, Kristensen S, Dao S, Diallo H, Diarra B, Noumsi TG, Guindo O. Seroprevalence of HIV/HBV coinfection in Malian blood donors. J Int Assoc Physicians AIDS Care (Chic Ill). 2009 Jan-Feb;8(1):47-51

10. Adewumi MO, Donbraye E, Sule WF, Olarinde O, Adewumi MO, Donbraye E, Sule WF, Olarinde O. HBV Infection Among HIV-Infected Cohort and HIV-Negative Hospital Attendees in South Western Nigeria. African Journal of Infectious Diseases. 2015 03/13/;9(1):14-7

11. Nwogoh B, Ikpomwen OD, Isoa EM. Donor blood procurement and the risk of transfusion transmissible viral infections in a tertiary health facility in South-South Nigeria. Nigerian Medical Journal. 2011 01/01/

12. Ajayi BB, Ajayi OD, Hamidu I, Dawurung JS, Ballah AD, Isah J, Chama CM. Seroprevalence of some sexually transmitted infections among antenatal attendees in university of Maiduguri teaching hospital, Maiduguri-Nigeria. Annals of Biological Research. 2013;4(2):141-5

13. Takalmawa HU, Emokpae MA, Abubakar AG, Kwaru AH. Prevalence of Hepatitis B Surface Antigen and human immunodeficiency virus antibodies among blood donors in Aminu Kano Teaching Hospital, Kano, Nigeria, 1996-2001. Hamdard Medicus. 2004;47(2):54-7

14. Mabayoje VO, Oparinde DP, Akanni EO, Taiwo SS, Muhibi MA, Adebayo TO. Seroprevalence of hepatitis B and C and of human immunodeficiency virus among blood donors in south-west Nigeria. British Journal of Biomedical Science. 2007;64(4):177-9

15. Oronsaye FE, Oronsaye JI. Prevalence of HIV-positives and hepatitis B surface antigen-positives among donors in the University of Benin Teaching Hospital, Nigeria. Tropical Doctor. 2004 Jul;34(3):159-60

16. Mbaawuaga EM. Studies on prevalence, co-infection and associated risk factors of hepatitis B virus (HBV) and human immunodeficiency virus (HIV) in Benue State, Nigeria. Sexually Transmitted Diseases. 2014 June;41:S143

17. Okonkwo UC, Okpara H, Otu A, Ameh S, Ogarekpe Y, Osim H, Inyama M. Prevalence of hepatitis B, hepatitis C and human immunodeficiency viruses, and evaluation of risk factors for transmission: report of a population screening in Nigeria. SAMJ - South African Medical Journal. 2017;107(4):346-51

18. Mavenyengwa RT, Moyo SR, Nordbo SA. Streptococcus agalactiae colonization and correlation with HIV-1 and HBV seroprevalence in pregnant women from Zimbabwe. Eur J Obstet Gynecol Reprod Biol. 2010 May;150(1):34-8

19. Dray X, Dray-Spira R, Bronstein JA, Mattera D. [Prevalences of HIV, hepatitis B and hepatitis C in blood donors in the Republic of Djibouti]. Med Trop (Mars). 2005;65(1):39-42

20. Tiruneh M. Seroprevalence of multiple sexually transmitted infections among antenatal clinic attendees in Gondar Health Center, northwest Ethiopia. Ethiop Med J. 2008 Oct;46(4):359-66

21. Yami A, Alemseged F, Hassen A. Hepatitis B and C Viruses Infections and Their Association with Human Immunodeficiency Virus: A Cross-Sectional Study among Blood Donors in Ethiopia. Ethiopian journal of health sciences. 2011 01/01/

22. Misganaw B. Prevalence of transfusion-transmissible infections in donors to an Ethiopian blood bank between 2009 and 2013 and donation factors that would improve the safety of the blood supply in underdeveloped countries. Laboratory Medicine. 2016;47(2):134-9

23. Biadgo B, Shiferaw E, Woldu B, Alene KA, Melku M. Transfusion-transmissible viral infections among blood donors at the North Gondar district blood bank, northwest Ethiopia: A three year retrospective study. PLoS ONE. 2017;12 (7) (no pagination)(e0180416)

24. Kerubo G, Khamadi S, Okoth V, Madise N, Ezeh A, Ziraba A, Mwau M. Hepatitis B, Hepatitis C and HIV-1 Coinfection in Two Informal Urban Settlements in Nairobi, Kenya.[Erratum appears in PLoS One. 2015;10(7):e0133342 Note: Abdalla, Ziraba [correctd to Ziraba, Abdhalah]; PMID: 26192604]. PLoS ONE [Electronic Resource]. 2015;10(6)

25. Cunha L, Plouzeau C, Ingrand P, Gudo JP, Ingrand I, Mondlane J, Beauchant M, Agius G. Use of replacement blood donors to study the epidemiology of major blood-borne viruses in the general population of Maputo, Mozambique. Journal of Medical Virology. 2007 Dec;79(12):1832-40

26. Stokx J, Gillet P, De Weggheleire A, Casas EC, Maendaenda R, Beulane AJ, Jani IV, Kidane S, Mosse CD, Jacobs J, Bottieau E. Seroprevalence of transfusion-transmissible infections and evaluation of the pre-donation screening performance at the Provincial Hospital of Tete, Mozambique. BMC Infectious Diseases. 2011;11:141

27. Ladner J, Leroy V, Simonon A, Karita E, Van de Perre P, Dabis F. Hepatitis B and HIV type 1 co-infection in pregnant African women. Médecine et Maladies Infectieuses. 2002 //;32(7):396-7

28. Bwogi J, Braka F, Makumbi I, Mishra V, Bakamutumaho B, Nanyunja M, Opio A, Downing R, Biryahwaho B, Lewis RF. Hepatitis B infection is highly endemic in Uganda: findings from a national serosurvey. African Health Sciences. 2009 Jun;9(2):98-108

29. Hladik W, Dollard SC, Downing RG, Kataaha P, Pellett PE, Karon JM, Mermin J, Lackritz EM. Kaposi's sarcoma in Uganda: risk factors for human herpesvirus 8 infection among blood donors. Journal of Acquired Immune Deficiency Syndromes: JAIDS. 2003 Jun 1;33(2):206-10

30. Boon D, Redd AD, Laeyendecker O, Engle RE, Nguyen H, Ocama P, Boaz I, Ndyanabo A, Kiggundu V, Reynolds SJ, Gray RH, Wawer MJ, Purcell RH, Kirk GD, Quinn TC, Stabinski L, Rakai Hlth Sci P. Hepatitis E Virus Seroprevalence and Correlates of Anti-HEV IgG Antibodies in the Rakai District, Uganda. Journal of Infectious Diseases. 2018 Mar;217(5):785-9

31. Stabinski L, Reynolds SJ, Ocama P, Laeyendecker O, Serwadda D, Gray RH, Wawer M, Thomas DL, Quinn TC, Kirk GD. Hepatitis B virus and sexual behavior in Rakai, Uganda. Journal of Medical Virology. 2011 May;83(5):796-800

32. Goncalez TTGON, Moreno ECM, Bolina-Santos BSE, Bruhn RB, Carneiro-Proietti ABFCP, Loureiro PL, Mendrone AM, Sabino ECS, Custer BC. HIV, hepatitis B, hepatitis C and HTLV co-infection among blood donors at three large Brazilian blood centers. Vox Sanguinis. 2015 June;Conference: 25th Regional Congress of the International Society of Blood Transfusion in Conjunction with the 33rd Annual Conference of the British Blood Transfusion Society. London United Kingdom. Conference Publication: (var.pagings). 109 (SUPPL. 1):229-30

33. Moura AA, de Mello MJ, Correia JB. Prevalence of syphilis, human immunodeficiency virus, hepatitis B virus, and human T-lymphotropic virus infections and coinfections during prenatal screening in an urban Northeastern Brazilian population. International Journal of Infectious Diseases. 2015 2015 Oct;39:10-5

34. Queiroz NMB, Sampaio DdA, Santos EdS, Bezerra ACdS. Logistic model for determining factors associated with HIV infection among blood donor candidates at the Fundação HEMOPE^ien. Rev bras hematol hemoter. 2012;34(3):217-21

35. Bedoya JAP, Marquez MMC, Arias JAC. Seroprevalence of markers of transfusion transmissible infections in blood bank in Colombia. Revista De Saude Publica. 2012 Dec;46(6):950-9

36. Sharma DC, Rai S, Bharat S, Iyenger S, Gupta S, Sao S, Jain B. Transfusion Transmissible Infections among Blood Donors at the Blood Bank of Medical College of Gwalior: A 5 Year Study. . 2014

37. Nayak S, Kakkar B, Bajpai M. Trends in the prevalence of transfusion transmitted infections among blood donors in a tertiary care hospital in north india: Eight years' experience. Vox Sanguinis. 2017 November;Conference: 28th Regional Congress of the International Society of Blood Transfusion. China. 112 (Supplement 2):93-4

38. Saini PA, Chakrabarti PR, Varma AV, Gambhir S, Tignath G, Gupta P. Hepatitis C virus: Unnoticed and on the rise in blood donor screening? A 5 years cross-sectional study on seroprevalence in voluntary blood donors from central India. Journal of Global Infectious Diseases. 2017 April-June;9 (2):51-5

39. Sawaithul VK, Ukey PM, Bobhate SK. Seroprevalence of HIV, HBV, HCV and Syphilis in blood donors of Central India. Biomedical Research. 2006 May/August;17(2):139-43

40. Bisht TV, Kanetkar SR, Kumbhar SS, Chavan S. Seroprevalence of infectious markers among blood donors from blood bank of a tertiary care hospital. Indian Journal of Hematology and Blood Transfusion. 2013 December;29 (4):391-2

41. Youssef M, Gamal A. Comparison between the prevalence of combinations of ttis and each parameter in voluntary non-remunerated blood donors. Vox Sanguinis. 2016 September;Conference: 34th International Congress of the International Society of Blood Transfusion. United Arab Emirates. 111 (Supplement 1):211-2

42. Fomulu NJ, Morfaw FLI, Torimiro JN, Nana P, Koh MV, William T. Prevalence, correlates and pattern of Hepatitis B among antenatal clinic attenders in Yaounde-Cameroon: Is perinatal transmission of HBV neglected in Cameroon? BMC Pregnancy and Childbirth. 2013 08 Aug;13:1-10

43. Beghin JC, Ruelle J, Sokal E, Bachy A, Krishna M, Hall L, Goubau P, Linden Dvd. Effectiveness of the South African expanded program of immunization against hepatitis B in children infected with human immunodeficiency virus-1 living in a resource-limited setting of KwaZulu-Natal. Journal of Medical Virology. 2017;89(1):182-5

44. Chotun N, Nel E, Cotton MF, Preiser W, Andersson MI. Hepatitis B virus infection in HIV-exposed infants in the Western Cape, South Africa. Vaccine. 2015 01/01/;33(36):4618-22

45. Muro FJ, Fiorillo SP, Sakasaka P, Odhiambo C, Reddy EA, Cunningham CK, Buchanan AM. Seroprevalence of hepatitis B and C viruses among children in Kilimanjaro Region, Tanzania. Journal of the Pediatric Infectious Diseases Society. 2013 01 Dec;2(4):320-6

46. Eke AC, Eke UA, Okafor CI, Ezebialu IU, Ogbuagu C. Prevalence, correlates and pattern of hepatitis B surface antigen in a low resource setting. Virology Journal. 2011;8:12

47. Shimelis T, Torben W, Medhin G, Tebeje M, Andualm A, Demessie F, Mulu A, Tegbaru B, Gebre-Selassie S. Hepatitis B virus infection among people attending the voluntary counselling and testing centre and anti-retroviral therapy clinic of St Paul's General Specialised Hospital, Addis Ababa, Ethiopia. Sex Transm Infect. 2008 Feb;84(1):37-41

48. Webale MK, Budambula V, Lihana R, Musumba FO, Nyamache AK, Budambula NLM, Ahmed AA, Ouma C, Were T. Hepatitis B virus sero-profiles and genotypes in HIV-1 infected and uninfected injection and Non-injection drug users from coastal Kenya.[Erratum appears in BMC Infect Dis. 2015;15:373 Note: Kilongosi, Mark W [corrected to Webale, Mark K]; PMID: 26334305]. BMC Infectious Diseases. 2015;15(299):30

49. Chimphambano C, Komolafe IO, Muula AS. Prevalence of HIV, HepBsAg and Hep C antibodies among inmates in Chichiri prison, Blantyre, Malawi. Malawi Medical Journal. 2007;19(3):107-10

50. Johnston LG, Corceal S. Unexpectedly high injection drug use, HIV and hepatitis C prevalence among female sex workers in the Republic of Mauritius. Aids Behav. 2013 Feb;17(2):574-84

51. Carobene M, Bolcic F, Farias MS, Quarleri J, Avila MM. HIV, HBV, and HCV molecular epidemiology among trans (transvestites, transsexuals, and transgender) sex workers in Argentina. Journal of Medical Virology. 2014 Jan;86(1):64-70

52. Portelinha Filho AM, Nascimento CUd, Tannouri TN, Troiani C, Ascêncio EL, Bonfim R, D'Andrea LAZ, Prestes-Carneiro LE. Seroprevalence of HBV, HCV and HIV co-infection in selected individuals from state of São Paulo, Brazil^ien. Mem Inst Oswaldo Cruz. 2009 11;104(7):960-3

53. Aires RS, Matos MAD, Lopes CLR, Teles SA, Kozlowski AG, Silva AMC, Filho JAA, Lago BV, Mello FCA, Martins RMB. Prevalence of hepatitis B virus infection among tuberculosis patients with or without HIV in Goiânia City, Brazil. Journal of clinical virology: the official publication of the Pan American Society for Clinical Virology. 2012 01/01/

54. Jose-Abrego A, Panduro A, Fierro NA, Roman S. High prevalence of HBV infection, detection of subgenotypes F1b, A2, and D4, and differential risk factors among Mexican risk populations with low socioeconomic status. Journal of Medical Virology. 2017;89(12):2149-57

55. Luis J, Felipe U, Patricia I, Veronica R, Yazmin M, Andrea G. Epidemiological characteristics of attendants of VCT in the context of a faster and integral diagnosis of HIV/STI in Condesa Clinic, Mexico City. Journal of the International AIDS Society. 2014 May;17:7

56. Weiss ES, Cornwell EE, 3rd, Wang T, Syin D, Millman EA, Pronovost PJ, Chang D, Makary MA. Human immunodeficiency virus and hepatitis testing and prevalence among surgical patients in an urban university hospital. Am J Surg. 2007 Jan;193(1):55-60

57. Desai Praseeda S, Anuradha D, Shastri Jayanthi S. A study on the HBV and the HCV infections in female sex workers and their co-infection with HIV. Journal of Clinical and Diagnostic Research. 2013 01 Feb;7(2):234-7

58. Roche R, Amrita S, Nayak R. Prevalence of the Human Immunodeficiency Virus, the Hepatitis B Virus and the Hepatitis C Virus among the Patients at a Tertiary Health Care Centre: A Five Year Study. Journal of Clinical and Diagnostic Research. 2012;6(4 [Suppl-2]):623-6

59. Jindal N, Arora U, Singh K. Prevalence of human immunodeficiency virus (HIV), hepatitis B virus, and hepatitis C virus in three groups of populations at high risk of HIV infection in Amritsar (Punjab), Northern India. Japanese Journal of Infectious Diseases. 2008 Jan;61(1):79-81

60. Nelwan EJ, Van Crevel R, Alisjahbana B, Indrati AK, Dwiyana RF, Nuralam N, Pohan HT, Jaya I, Meheus A, Van Der Ven A. Human immunodeficiency virus, hepatitis B and hepatitis C in an Indonesian prison: prevalence, risk factors and implications of HIV screening. Tropical Medicine & International Health. 2010 Dec;15(12):1491-8

61. Paris R, Sirisopana N, Benenson M, Amphaiphis R, Tuntichaivanich C, Myint KS, Brown AE. The association between hepatitis C virus and HIV-1 in preparatory cohorts for HIV vaccine trials in Thailand. AIDS. 2003 Jun 13;17(9):1363-7

62. Nguyen CH, Ishizaki A, Chung PT, Hoang HT, Nguyen TV, Tanimoto T, Lihana R, Matsushita K, Bi X, Pham TV, Ichimura H. Prevalence of HBV infection among different HIV-risk groups in Hai Phong, Vietnam. Journal of Medical Virology. 2011 Mar;83(3):399-404

63. O'Connell S, Lillis D, Cotter A, O'Dea S, Tuite H, Fleming C, Crowley B, Fitzgerald I, Dalby L, Barry H, Shields D, Norris S, Plunkett PK, Bergin C. Opt-out panel testing for HIV, hepatitis B and hepatitis C in an urban emergency department: a pilot study. PLoS ONE. 2016;11(3):e0150546

64. Rivas P, Herrero MD, Poveda E, Madejon A, Trevino A, Gutierrez M, de Guevara CL, Lago M, de Mendoza C, Soriano V, Puente S. Hepatitis B, C, and D and HIV Infections among Immigrants from Equatorial Guinea Living in Spain. American Journal of Tropical Medicine and Hygiene. 2013 Apr;88(4):789-94

65. Zhang T, He N, Ding Y, Crabtree K, Minhas V, Wood C. Prevalence of human herpesvirus 8 and hepatitis C virus in a rural community with a high risk for blood-borne infections in central China. Clinical Microbiology & Infection. 2011 Mar;17(3):395-401

66. Hsieh M, Tsai J, Hsieh M, Huang C, Yeh M, Yang J, Chang K, Lin W, Lin C, Chen T, Huang J, Dai C, Yu M, Chuang W. Hepatitis C virus infection among injection drug users with and without human immunodeficiency virus co-infection. PLoS ONE. 2014;9(4)

67. Hsieh MH, Hsieh MY, Huang CF, Yeh ML, Wang SC, Yang JF, Chang K, Lin WR, Lin CY, Chen TC, Huang JF, Dai CY, Tsai JJ, Chuang WL, Yu ML. Anti-HIV seropositivity was related to HBsAg seropositivity among injecting drug users in Taiwan. Kaohsiung Journal of Medical Sciences. 2016 01;32 (2):96-102

68. Ray Saraswati L, Sarna A, Sebastian MP, Sharma V, Madan I, Thior I, Pulerwitz J, Tun W. HIV, Hepatitis B and C among people who inject drugs: high prevalence of HIV and Hepatitis C RNA positive infections observed in Delhi, India. BMC Public Health. 2015;15(1):726-

69. Zhou YH, Liu FL, Yao ZH, Duo L, Li H, Sun Y, Zheng YT. Comparison of HIV-, HBV-, HCV- and co-infection prevalence between Chinese and Burmese intravenous drug users of the China-Myanmar border region. PLoS ONE [Electronic Resource]. 2011;6(1):e16349

70. Nadol P, O'Connor S, Duong H, Le L-VN, Thang PH, Tram TH, Ha HTT, McConnell MS, Partridge J, Kaldor J, Law M, Nguyen TA. Findings from integrated behavioral and biologic survey among males who inject drugs (MWID) - Vietnam, 2009-2010: evidence of the need for an integrated response to HIV, hepatitis B virus, and hepatitis C virus. PLoS ONE [Electronic Resource]. 2015;10(2)

71. Ishizaki A, Tran VT, Nguyen CH, Tanimoto T, Hoang HTT, Pham HV, Phan CTT, Bi XQ, Pham TV, Ichimura H. Discrepancies in prevalence trends for HIV, hepatitis B virus, and hepatitis C virus in Haiphong, Vietnam from 2007 to 2012. Plos One. 2017 Jun;12(6)

72. Mir-Nasseri MM, Mohammadkhani A, Tavakkoli H, Ansari E, Poustchi H. Incarceration is a major risk factor for blood-borne infection among intravenous drug users. Hepatitis Monthly. 2011 January;11(1):19-22

73. Alavi SM, Etemadi A. HIV/HBV, HIV/HCV and HIV/HTLV-1 co infection among injecting drug user patients hospitalized at the infectious disease ward of a training hospital in Iran. Pakistan Journal of Medical Sciences. 2007 July/September;23(4):510-3

74. Mirzoyan L, Berendes S, Jeffery C, Thomson J, Ben Othman H, Danon L, Turki AA, Saffialden R, Valadez JJ. New Evidence on the HIV Epidemic in Libya: Why Countries Must Implement Prevention Programs Among People Who Inject Drugs. Jaids-Journal of Acquired Immune Deficiency Syndromes. 2013 Apr;62(5):577-83

75. Li JR, Gong RY, Tian KL, Wang J, Wang YX, Huang HJ. Study on the blood-borne virus co-infection and T lymphocyte subset among intravenous drug users. World Journal of Gastroenterology. 2007 Apr 28;13(16):2357-62

76. Zhou YH, Yao ZH, Liu FL, Li H, Jiang L, Zhu JW, Zheng YT. High prevalence of HIV, HCV, HBV and co-infection and associated risk factors among injecting drug users in Yunnan Province, China. PLoS ONE. 2012 16 Aug;7(8)

77. Chu FY, Chiang SC, Su FH, Chang YY, Cheng SH. Prevalence of human immunodeficiency virus and its association with hepatitis B, C, and D virus infections among incarcerated male substance abusers in Taiwan. Journal of Medical Virology. 2009 Jun;81(6):973-8

78. Dahoma M, Johnston LG, Holman A, Miller LA, Mussa M, Othman A, Khatib A, Issa R, Kendall C, Kim AA. HIV and related risk behavior among men who have sex with men in Zanzibar, Tanzania: results of a behavioral surveillance survey. Aids Behav. 2011 Jan;15(1):186-92

79. Sutmoller F, Penna TL, de Souza CT, Lambert J, Oswaldo Cruz Foundation STDHIVPG. Human immunodeficiency virus incidence and risk behavior in the 'Projeto Rio': results of the first 5 years of the Rio de Janeiro open cohort of homosexual and bisexual men, 1994-98. International Journal of Infectious Diseases. 2002 Dec;6(4):259-65

80. Lama JR, Agurto HS, Guanira JV, Ganoza C, Casapia M, Ojeda N, Ortiz A, Zamalloa V, Suarez-Ognio L, Cabezas C, Sanchez JL, Sanchez J, Peruvian HIVSSWG. Hepatitis B infection and association with other sexually transmitted infections among men who have sex with men in Peru. Am J Trop Med Hyg. 2010 Jul;83(1):194-200

81. Remis RS, Liu J, Loutfy MR, Tharao W, Anuradha R, Huibner S, Kesler M, Halpenny R, Grennan T, Brunetta J, Smith G, Reko T, Kaul R. Prevalence of sexually transmitted viral and bacterial infections in HIV-positive and HIV-negative men who have sex with men in Toronto. PLoS ONE. 2016;11(7):e0158090

82. Price H, Bansi L, Sabin CA, Bhagani S, Burroughs A, Chadwick D, Dunn D, Fisher M, Main J, Nelson M, Pillay D, Rodger A, Taylor C, Gilson R. Hepatitis B Virus Infection in HIV-Positive Individuals in the UK Collaborative HIV Cohort (UK CHIC) Study. PLoS ONE. 2012 07 Nov;7(11)

83. Kojima Y, Kawahata T, Mori H, Furubayashi K, Taniguchi Y, Iwasa A, Taniguchi K, Kimura H, Komano J. Prevalence and epidemiological traits of HIV infections in populations with high-risk behaviours as revealed by genetic analysis of HBV. Epidemiology & Infection. 2013 Nov;141(11):2410-7
